# Study specific prediction intervals for random‐effects meta‐analysis: A tutorial: Prediction intervals in meta‐analysis

**DOI:** 10.1002/jrsm.1490

**Published:** 2021-06-03

**Authors:** Robbie C. M. van Aert, Christopher H. Schmid, David Svensson, Dan Jackson

**Affiliations:** ^1^ Methodology and Statistics Tilburg University Tilburg Netherlands; ^2^ Department of Biostatistics Brown University Providence Rhode Island USA; ^3^ Statistical Innovation Cambridge UK

**Keywords:** best linear unbiased prediction, empirical Bayes estimate, forest plot, prediction interval, shrinkage

## Abstract

The pooled estimate of the average effect is of primary interest when fitting the random‐effects model for meta‐analysis. However estimates of study specific effects, for example those displayed on forest plots, are also often of interest. In this tutorial, we present the case, with the accompanying statistical theory, for estimating the study specific true effects using so called 'empirical Bayes estimates' or 'Best Unbiased Linear Predictions' under the random‐effects model. These estimates can be accompanied by prediction intervals that indicate a plausible range of study specific true effects. We coalesce and elucidate the available literature and illustrate the methodology using two published meta‐analyses as examples. We also perform a simulation study that reveals that coverage probability of study specific prediction intervals are substantially too low if the between‐study variance is small but not negligible. Researchers need to be aware of this defect when interpreting prediction intervals. We also show how empirical Bayes estimates, accompanied with study specific prediction intervals, can embellish forest plots. We hope that this tutorial will serve to provide a clear theoretical underpinning for this methodology and encourage its widespread adoption.

## 1 INTRODUCTION

The random‐effects model^1^, ^2^, ^3^, ^4^, ^5^, ^6^ is routinely used in meta‐analyses. This model allows us to make inferences about the average effect whilst relaxing the usually strong, and so difficult to defend, assumption that all studies estimate a common treatment effect. The random‐effects model achieves this by incorporating a between‐study variance parameter. This parameter quantifies the variation in the studies' estimated effects that is not explained by within‐study variation alone. A wide variety of estimators for the between‐study variance, and corresponding methods for calculating confidence intervals, have been proposed.^7^, ^8^ Statistical tests for the absence of between‐study heterogeneity,^9^, ^10^ and statistics that quantify its impact,^11^ are also available.

Despite this variety of statistical methods relating to between‐study heterogeneity, formal statistical inference usually focuses on the average effect. In conventional meta‐analysis methodologies, the uncertainty in the estimation of the between‐study variance is ignored when making inferences about this parameter. By further ignoring the uncertainty in the within‐study variances throughout the analysis, simple statistical methods can then be used. A consequence of ignoring the uncertainty in the variance components is that inferences from conventional meta‐analysis are merely approximate, because all uncertainty in the estimated variance components has been ignored,^12^ which may yield inaccurate statistical inferences.^13^, ^14^, ^15^, ^16^

Inferences for the average effect usually include point estimates, standard errors, confidence intervals, and the results from hypothesis tests. Routinely reporting prediction intervals for the true effect in a new study has also been proposed.^5^, ^6^ These prediction intervals provide insight into the extent of the between‐study heterogeneity and are intended to accompany confidence intervals for the pooled effect. This is especially of importance for clinical practice and decision making, because this type of prediction interval may, for example, clearly convey that a treatment or drug is efficacious in some studies but not in others.

Although the random‐effects model only includes two parameters (the average effect and the between‐study variance) it also includes an unobserved random‐effect for each study. This means that every study is assumed to estimate a different true underlying effect. Study specific estimated effects are conventionally displayed, with their confidence intervals, on forest plots.^17^ Only within‐study information is used when computing these estimates that are identical, and valid, under both the common‐effect (between‐study variance taken to be zero) and random‐effects models. This is in contrast to the statistical inferences for the average effect discussed above, that make extensive use of the assumptions of the random‐effects model^12^ and information from all studies. These observations beg the question, can the assumptions of the random‐effects model be used further to produce (in some senses) better, or even just alternative, study specific effect size estimates and corresponding intervals?

The answer to this question is 'yes'. By assuming the random‐effects model, we can justify the use of so called 'empirical Bayes estimates' or 'Best Linear Unbiased Predictions' (BLUPs) of the study specific true effects.^18^ These are linear combinations of the conventional study specific estimated effects and the estimated average effect, reflecting the intuition that estimates from other studies add information to any specific study's true effect. That is, a study specific true effect estimate '*borrows strength*' from the other studies in the meta‐analysis to obtain an estimate closer to the underlying true value. These study specific true effects are of interest, because a meta‐analyst may want to know what the true effect is in a particular study population. These populations could, for example, correspond to a specific country, type of patient, or drug dosage; study specific true effects allow drawing inferences for studies with these particular characteristics. A study specific true effect could also be used when designing a new study, for example, when computing the required sample size using a statistical power analysis.

It is sometimes argued that this approach provides a better indication of a study's effect than the more familiar study specific estimates conventionally displayed on forest plots.^19^, ^20^ Although these ideas are not new, the literature on this topic is diffuse, and does not always use consistent terminologies and statistical justifications.^21^, ^22^, ^23^, ^24^ Furthermore, different variance formulae for computing study specific prediction intervals are available; by examining these formulae, we are able to emphasize some rather subtle considerations that might otherwise be overlooked, or worse result in mistakes. The main aims of this tutorial are to distill the relevant literature, and so make it accessible to a wider audience, and unify empirical Bayes estimates and BLUPs by showing that these are the same under the random‐effects meta‐analysis model. Another aim of this tutorial is to make recommendations for best practice. One concrete, but not novel, recommendation is that we advise embellishing forest plots by using the proposed alternative estimators of the study specific effects. In addition, we enhance this tutorial by undertaking a simulation study to critically evaluate the performance of the proposed methodology. We hope that this tutorial will lead to widespread use of the methods we discuss, whilst simultaneously raising awareness of their limitations.

The rest of this tutorial is set out as follows. In section 2, we present the random‐effects model using several, but equivalent, representations. We use the second and third of these representations to derive the empirical Bayes estimates and the BLUPs as the same numerical quantities. In section 3, we derive three variance formulae for the empirical Bayes estimates and BLUPs, and present the case for one of these being the preferred approach. In section 4, we show how forest plots can be embellished if estimates of the study specific true effects and their prediction intervals are added. In section 5, we enhance this tutorial with original research by examining the statistical properties of study specific prediction intervals using a simulation study. We end with a discussion section in section 6.

## 2 THE RANDOM‐EFFECTS MODEL

The random‐effects model can be represented in three equivalent ways. This model uses normal approximations for the estimated effects. We return to the possibility of using more advanced models, such as generalized linear mixed models, in the discussion. The hierarchical representation will probably be the most familiar, so we start by introducing it. We will then present the less commonly used bivariate representation, because this will be necessary to derive empirical Bayes estimates. Finally, we will present the variance components representation, because this will be necessary to derive the BLUPs as the same numerical quantities.

### 2.1 The hierarchical representation

To allow for between‐study heterogeneity, the random‐effects model assumes that the study specific true effects *θ*_*i*_, *i* = 1, 2, ⋯*n*, are normally distributed, centered at the overall average effect *θ* with between‐study variance *τ* ^2^. The random‐effects model then further assumes, given *θ*_*i*_, that the estimated effect *Y*_*i*_ from the *i*th study is normally distributed with mean *θ*_*i*_. The variance of *Y*_*i*_ in this conditional distribution is usually referred to as a within‐study variance and can be estimated for a wide variety of outcome measures.^25^, ^26^ Conventional methods for meta‐analysis treat the within‐study variances as fixed and known and we also adopt this convention throughout the tutorial. Hence, we assume that $Y_{i}|\theta_{i}∼N\left(\theta_{i},\sigma_{i}^{2}\right)$ under the random‐effects model. The *Y*_*i*_ are conventionally displayed on forest plots with their confidence intervals [*Y*_*i*_ − 1.96*σ*_*i*_, *Y*_*i*_ + 1.96*σ*_*i*_] where 1.96 refers to the 1 − *α*/2 quantile of the standard normal distribution with *α* = 0.05.

We also assume that studies are independent, so that all random variables associated with the *i*th study are independent of those associated with the *j*th study *i* ≠ *j*. We call this the hierarchical representation, where the models for *Y*_*i*_|*θ*_*i*_ and *θ*_*i*_ are the first level, and second level, in the hierarchical model, respectively. If *τ* ^2^ = 0 then the random‐effects model collapses to the common‐effect model.

#### 2.1.1 Making inferences

The joint probability density function of the true study specific effect *θ*_*i*_ and the estimated effect *Y*_*i*_ from the *i*th study is given by the product of the probability densities of the two normal distributions in the hierarchical model, that is. *f*(*θ*_*i*_, *Y*_*i*_) = *f*(*θ*_*i*_)*f*(*Y*_*i*_|*θ*_*i*_). The *θ*_*i*_ are usually regarded as nuisance parameters and can be integrated out of this joint probability density function to give the marginal density of *Y*_*i*_. Elementary calculations provide the marginal distribution $Y_{i}∼N\left(\theta ,\sigma_{i}^{2}+\tau^{2}\right)$. Inferences for *θ* and *τ* ^2^ can then be made using this marginal distribution.

A wide variety of estimators of *τ* ^2^ are possible.^7^, ^8^ Upon estimating *τ* ^2^, the conventional method for making inferences about *θ* approximates the true *τ* ^2^ with its estimate ${\hat{\tau }}^{2}$. Hence, when making these inferences the *Y*_*i*_ are modeled as normally distributed random variables, centered at *θ* with different (but 'known') variances $\sigma_{i}^{2}+{\hat{\tau }}^{2}$. The inferences for *θ* therefore require only calculations using the normal distribution. Specifically, writing ${\hat{w}}_{i}={\left(\sigma_{i}^{2}+{\hat{\tau }}^{2}\right)}^{-1}$, we have that

(1) $$\hat{\theta }=\sum {\hat{w}}_{i}Y_{i}/\sum {\hat{w}}_{i}$$

where $\hat{\theta }∼N\left(\theta ,1/\sum {\hat{w}}_{i}\right)$. By integrating out the *θ*_*i*_ in this way, however, we do not immediately obtain inferences for the *θ*_*i*_. In order to motivate methods for estimating these random‐effects we require alternative representations of the random‐effects model.

### 2.2 The bivariate representation

The hierarchical model $Y_{i}|\theta_{i}∼N\left(\theta_{i},\sigma_{i}^{2}\right)$ and *θ*_*i*_ ∼ *N*(*θ*, *τ* ^2^) described in Section 2.1 is equivalent to

(2) $$\left[\begin{array}{c} \theta_{i}\\ Y_{i} \end{array}\right]∼N\left(\left[\begin{array}{c} \theta \\ \theta \end{array}\right],\left[\begin{array}{cc} \tau^{2} & \tau^{2}\\ \tau^{2} & \sigma_{i}^{2}+\tau^{2} \end{array}\right]\right)$$

as can be seen by noting that the joint distribution gives the correct marginal distributions *θ*_*i*_ ∼ *N*(*θ*, *τ* ^2^) and $Y_{i}∼N\left(\theta ,\sigma_{i}^{2}+\tau^{2}\right)$, and also the correct conditional distribution $Y_{i}|\theta_{i}∼N\left(\theta_{i},\sigma_{i}^{2}\right)$. The marginal distributions are immediately obvious from the model in (2), and the conditional distribution can be obtained using standard formulae (e.g., result 4.6 in Johnson and Wichern^27^) for deriving the conditional distributions from a multivariate normal distribution. We call this the bivariate representation, because a bivariate normal is used to describe the joint distribution of *θ*_*i*_ and *Y*_*i*_.

From elementary calculations using standard properties of the bivariate normal distribution, the conditional distribution of *θ*_*i*_|*Y*_*i*_ from model (2) is

(3) $$\theta_{i}|Y_{i};\theta ,\tau^{2}∼N\left(B_{i}\theta +\left(1-B_{i}\right)Y_{i},\sigma_{i}^{2}\left(1-B_{i}\right)\right)$$

where $B_{i}=\sigma_{i}^{2}/\left(\sigma_{i}^{2}+\tau^{2}\right)$ and we now place *θ*, *τ* ^2^ after a semicolon to emphasize the dependence of this conditional distribution on the model parameters. Equation (3) is also the conditional distribution of *θ*_*i*_ given **Y** and the model parameters, where **Y** is the *n* × 1vector containing the *Y*_*i*_. This is because all random variables associated with the *i*th study are independent of those associated with the *j*th study, *i* ≠ *j*.

The parameters of the conditional normal distributions in Equation (3) are functions of *θ* and *τ* ^2^, where their dependence on *τ* ^2^ is via *B*_*i*_. To evaluate Equation (3) we require the values of both unknown parameters. If we replace these parameters with their estimates, then the mean of the normal distribution in Equation (3) becomes

(4) $${\hat{\theta }}_{i}={\hat{B}}_{i}\hat{\theta }+\left(1-{\hat{B}}_{i}\right)Y_{i}$$

where ${\hat{B}}_{i}=\sigma_{i}^{2}/\left(\sigma_{i}^{2}+{\hat{\tau }}^{2}\right)$ and $\hat{\theta }$ is given in Equation (1). We use the notation ${\hat{\theta }}_{i}$ in Equation (4), because this is the empirical Bayes Estimator^18^, ^28^ of *θ*_*i*_. The estimate *θ*_*i*_ is our proposed alternative to the conventional study specific estimate *Y*_*i*_, that allows information from all studies to inform inferences about the study specific true effects under the random‐effects model. Note that Equation (4) illustrates that the estimate of the study specific true effect ${\hat{\theta }}_{i}$ becomes closer (i.e., shrinks more) to $\hat{\theta }$ as ${\hat{\tau }}^{2}$ decreases. We have now established the first justification for the point estimates of the study specific *θ*_*i*_ that provide our focus. A related idea can be found in Rücker et al.'s^29^ Equation (2.6) where, in the context of limit meta‐analysis, study effects are shrunk towards a new study effect that are (unlike here) also adjusted for small‐study effects.

### 2.3 The variance components representation

We can also write the random‐effects model in variance components form.^4^, ^30^, ^31^ When using this representation, we parameterize the estimated effect in the *i*th study as

(5) $$Y_{i}=\theta +\gamma_{i}+{ɛ}_{i}$$

where *γ*_*i*_ ∼ *N*(0, *τ* ^2^) and ${ɛ}_{i}∼N\left(0,\sigma_{i}^{2}\right)$ and all *γ*_*i*_ and *ɛ*_*i*_ are independent. The true effect of the *i*th study is then

(6) $$\theta_{i}=\theta +\gamma_{i}.$$

The random‐effect *γ*_*i*_ is therefore the amount that the true effect in the *i*th study deviates from the overall average effect *θ*. We call this the variance components representation because the quantities that model the variation in the data are represented as two separate components in Equation (5).

Robinson^32^ and Laird and Ware^33^ consider a more general model and we use Robinson's^32^ notation for describing this model. The more general model is

Y=Xβ+Zu+e

where ***β*** is a column vector of unknown parameters (fixed effects), **X** and **Z** are known matrices and **u** and **e** are column vectors containing unobservable random variables (random‐effects). Robinson further assumes that *E*[**u**] = *E*[**e**] = **0** and

$$\mathrm{Var}\left[\begin{array}{c} u\\ e \end{array}\right]=\left[\begin{array}{cc} G & 0\\ 0 & R \end{array}\right]\tau^{2}$$

where *τ* ^2^ is a positive constant (Robinson^32^ uses *σ* ^2^ for this constant, but we have equated his *σ* ^2^ with our *τ* ^2^ to avoid a clash of notation).

The random‐effects model for meta‐analysis is then obtained by considering a special case of this more general model. Taking **X** = **1** (the column vector of length *n* where every entry is one), **Z** = **G** = **I** (the *n* × *n* identity matrix), we obtain the random‐effects model for meta‐analysis where ***β*** = *θ*, **u** is the column vector of length *n* containing the *γ*_*i*_, and **R** = diag $\left(\sigma_{i}^{2}/\tau^{2}\right)$ (the *n* × *n* diagonal matrix containing the ratios of the $\sigma_{i}^{2}$ and *τ* ^2^). The common‐effect model (*τ* ^2^ = 0) for meta‐analysis is obtained by taking the limit *τ* ^2^ → 0^+^; the definition of **R** = diag $\left(\sigma_{i}^{2}/\tau^{2}\right)$ ensures that all studies have the correct within‐study variance $\sigma_{i}^{2}$ for all *τ* ^2^, so that we obtain the common‐effect model in the limit *τ* ^2^ → 0^+^.

After making the simplifications in the previous paragraph, in the web Supplementary materials we show that the second equation in (1.2) of Robinson^32^ then gives the BLUP for *γ*_*i*_ in terms of $\hat{\theta }$ and is estimated as

$${\hat{\gamma }}_{i}=\left(1-B_{i}\right)\left(Y_{i}-\hat{\theta }\right).$$

From model (6), we then obtain ${\hat{\theta }}_{i}=\hat{\theta }+{\hat{\gamma }}_{i}$, where $\hat{\theta }$ is given in Equation (1). Upon further replacing *τ* ^2^ with its estimate in the definition of *B*_*i*_, this immediately results in Equation (4).

We therefore conclude that (ignoring the uncertainty in the $\sigma_{i}^{2}$ and *τ* ^2^) our estimate ${\hat{\theta }}_{i}$ of the study specific true effect in the *i*th study, given in Equation (4), has a dual interpretation: it is both the empirical Bayes estimate and the BLUP. In making this second statement, it is perhaps pertinent to state what is meant by the term 'BLUP'. The BLUP is the 'B'est because var(${\hat{\theta }}_{i}-\theta_{i})$ is as small as possible; 'L'inear because it is linear in the *Y*_*i*_; 'U'nbiased in the sense that E(${\hat{\theta }}_{i}-\theta_{i})=0$; and it is said to be a 'P'rediction because it relates to an unobservable random‐effect. Under the normality assumptions made, we may omit the 'L' for linear, because linear estimates are also the best under these assumptions. An important conceptual difference between these two interpretations is that the empirical Bayes interpretation involves a form of Bayesian reasoning whereas the BLUP does not. Moreover, empirical Bayes requires a normality assumption whereas such a normality assumption is not needed for the BLUP.

### 2.4 Recommendation

We propose presenting the ${\hat{\theta }}_{i}$, for example on forest plots,^17^ on the basis that they are both the empirical Bayes estimates and the BLUPs given the normality assumptions we have made. However, we propose presenting these estimates together with corresponding intervals in addition to, and never instead of, the *Y*_*i*_. We suggest continuing to use the term 'confidence interval' for the conventional intervals already shown on forest plots with the *Y*_*i*_, and using the alternative term 'study specific prediction interval' for those that accompany the ${\hat{\theta }}_{i}$. This follows the convention^32^ of using the term 'prediction' when estimating random variables, but 'model based confidence interval' is an alternative term that some might prefer. A variance formula is needed to compute these intervals, and we address this issue in the next section.

## 3 VARIANCE FORMULAE FOR COMPUTING STUDY SPECIFIC PREDICTION INTERVALS

We have now justified the proposed point estimates ${\hat{\theta }}_{i}$ in two different ways. With a suitable variance formula, study specific prediction intervals can be calculated to accompany them. By continuing to ignore the uncertainty in the estimated within and between‐study variances, the ${\hat{\theta }}_{i}$ are normally distributed, so that quantiles from the standard normal distribution can be used to compute these prediction intervals.

We now describe three possible variance formulae, and discuss their derivations and relative merits.

### 3.1 Crude formula

The simplest possibility is to conceptualize ${\hat{\theta }}_{i}$ as the empirical Bayes estimator, and use the variance from Equation (3). This results in the crude formula

(7) $$\mathrm{Var}\left({\hat{\theta }}_{i}|Y_{i};\theta ,\tau^{2}\right)=\sigma_{i}^{2}\left(1-B_{i}\right)=\sigma_{i}^{2}\tau^{2}/\left(\sigma_{i}^{2}+\tau^{2}\right)$$

where we further replace *τ* ^2^ with its estimate ${\hat{\tau }}^{2}$ to estimate this variance in practice. This is the variance of *θ*_*i*_, given *Y*_*i*_, if we ignore all parameter uncertainty in both *θ* and *τ* ^2^. However, this parameter uncertainty is usually considerable in practice, so this 'plugin' formula cannot possibly be regarded as anything other than crude. Furthermore if ${\hat{\tau }}^{2}=0$, which is a common occurrence in practice, then this formula with this point estimate gives a variance of zero which is, at best, an undesirable property. A further concern is that, because this variance follows from a Bayesian argument, the repeated sampling properties of the resulting interval are not necessarily clear in a frequentist analysis. Despite this, we present this formula because it provides a useful 'stepping stone' to the next one.

Note that by taking the reciprocal of Equation (7), which requires *τ* ^2^ > 0 to avoid division by zero, we can see that the corresponding precision of ${\hat{\theta }}_{i}$, given *Y*_*i*_ and the model parameters, is equal to the sum of the within‐study precision ($1/\sigma_{i}^{2}$) and the between‐study precision (1/*τ* ^2^). Working in terms of the precision is therefore less opaque, but ultimately variance formulae are needed to compute prediction intervals.

### 3.2 Raudenbush formula

One way to improve the first variance formula above is to take into account the uncertainty in *θ* (but ignore the uncertainty in *τ* ^2^, as we do when making inferences about *θ* in conventional meta‐analysis). To derive our second variance formula, we will now employ fully Bayesian reasoning,^34^ and so in this section we treat *θ* and *τ* ^2^ as random variables. However, we continue to adopt the usual convention of treating the $\sigma_{i}^{2}$ as fixed and known constants.

In a fully Bayesian context, Equation (3) gives the posterior mean and variance of *θ*_*i*_ given the data *Y*_*i*_, and also *θ* and *τ* ^2^. Hence, we now remove the semicolon from Equation (3) and treat *θ* and *τ* ^2^ as random variables. Upon assuming a suitable posterior distribution for *θ* (given *τ* ^2^), we can derive the posterior variance of *θ*_*i*_ given only the data and *τ* ^2^, using the usual law of total variance, Var(*Y*) = E_*X*_[Var(*Y* |*X*)] + Var_*X*_[E(*Y* |*X*)].

An approximate posterior distribution for *θ* that will be used in this calculation below is

(8) $$\theta |\left(Y,\tau^{2}\right)∼N\left(\mu_{\theta },1/\sum {\left(\sigma_{i}^{2}+\tau^{2}\right)}^{-1}\right)$$

where *μ*_*θ*_ is the expectation of the posterior distribution of *θ*. This approximate posterior distribution for *θ* in Equation (8) will be sufficiently accurate provided that a vague prior for *θ* is used and the sample size is reasonably large, so that the precision of classical and Bayesian estimates of *θ* are similar. We then have

$$\mathrm{Var}\left(\theta_{i}|Y,\tau^{2}\right)=E_{\theta |\left(Y,\tau^{2}\right)}\left[\mathrm{Var}\left(\theta_{i}|Y,\theta ,\tau^{2}\right)\right]+{\mathrm{Var}}_{\theta |\left(Y,\tau^{2}\right)}\left[E\left(\theta_{i}|Y,\theta ,\tau^{2}\right)\right]$$

so that from Equation (3), where this equation describes *θ*_*i*_|(*Y*_*i*_, *θ*, *τ* ^2^) as well as the equivalent *θ*_*i*_|(**Y**, *θ*, *τ* ^2^) in the fully Bayesian context,

(9) $$\mathrm{Var}\left(\theta_{i}|Y,\tau^{2}\right)=E_{\theta |\left(Y,\tau^{2}\right)}\left[\sigma_{i}^{2}\left(1-B_{i}\right)\right]+{\mathrm{Var}}_{\theta |\left(Y,\tau^{2}\right)}\left[B_{i}\theta +\left(1-B_{i}\right)Y_{i}\right].$$

Noting that, given (**Y**, *τ* ^2^), *B*_*i*_, *τ* ^2^ and (crucially) *Y*_*i*_ are constants, from the definition of *B*_*i*_ and the approximate posterior variance of *θ* in Equation (8), Equation (9) becomes

(10) $$\mathrm{Var}\left(\theta_{i}|Y,\tau^{2}\right)=\frac{\sigma_{i}^{2}\tau^{2}}{\sigma_{i}^{2}+\tau^{2}}+\frac{1}{w}\frac{\sigma_{i}^{4}}{{\left(\sigma_{i}^{2}+\tau^{2}\right)}^{2}}$$

where $w=\sum w_{i}=\sum {\left(\sigma_{i}^{2}+\tau^{2}\right)}^{-1}$. We ignore the uncertainty in the estimated between‐study variance, and so replace *τ* ^2^ with its estimate when computing the variance in Equation (10). Note that 1/*w* in Equation (10) is the variance of $\hat{\theta }$ in the random‐effects model.

We name the formula in (10) the Raudenbush formula, because it is a special case of the more general result (A6 of Raudenbush and Bryk^18^). Comparing the estimation equations for the variance in (10) and (7), we can see that Equation (10) introduces an additional term with *w* in the denominator. We therefore obtain Equation (7) from (10) in the limit as the number of studies, and so *w*, tends towards infinity. Furthermore, Equation (10) is simply 1/*w* when *τ* ^2^ = 0, so we obtain the variance from the common‐effect model (*τ* ^2^ = 0) when the point estimate ${\hat{\tau }}^{2}=0$ is substituted into (10). From Equations (1) and (3), ${\hat{\theta }}_{i}$ is the common effect estimate of *θ* when *τ* ^2^ = 0. Hence, inferences for *θ*_*i*_ are the same as those for *θ* when *τ* ^2^ = 0 which is entirely appropriate under the common‐effect model. In summary, the variance in (10) is an intuitively appealing variance to use. This formula is used by the R package metafor^35^ to create study specific prediction intervals in a meta‐analysis upon replacing *τ* ^2^ with its estimate.

#### 3.2.1 An alternative justification for the Raudenbush formula

We can also derive Equation (10) as the variance of $\left({\hat{\theta }}_{i}-\theta_{i}\right)$ without requiring Bayesian reasoning. To do so, we now interpret ${\hat{\theta }}_{i}$ as the BLUP, and calculate prediction intervals for *θ*_*i*_ using the pivot

(11) $$\left({\hat{\theta }}_{i}-\theta_{i}\right)∼N\left(0,V_{i}\right)$$

where *V*_*i*_ is to be calculated. When computing *V*_*i*_, the uncertainty in *τ* ^2^ is ignored and this parameter is treated as fixed and known.

Then

(12) $$V_{i}=\mathrm{Var}\left({\hat{\theta }}_{i}-\theta_{i}\right)=\mathrm{Var}\left({\hat{\theta }}_{i}\right)+\mathrm{Var}\left(\theta_{i}\right)-2\mathrm{Cov}\left({\hat{\theta }}_{i},\theta_{i}\right).$$

In the web Supplementary materials we show that

(13) $$\mathrm{Var}\left({\hat{\theta }}_{i}\right)=\frac{\tau^{4}}{\sigma_{i}^{2}+\tau^{2}}+\frac{1}{w}\frac{\sigma_{i}^{2}\left(\sigma_{i}^{2}+2\tau^{2}\right)}{{\left(\sigma_{i}^{2}+\tau^{2}\right)}^{2}},$$

(14) $$\mathrm{Var}\left(\theta_{i}\right)=\tau^{2}$$

and

(15) $$-2\mathrm{Cov}\left({\hat{\theta }}_{i},\theta_{i}\right)=-\frac{2}{w}\frac{\sigma_{i}^{2}\tau^{2}}{{\left(\sigma_{i}^{2}+\tau^{2}\right)}^{2}}-\frac{2\tau^{4}}{\sigma_{i}^{2}+\tau^{2}}.$$

Substituting Equations (13), (14), and (15) into Equation (12), and a little algebra yields the same variance as in Equation (10). We therefore now have a second justification for using this variance formula to compute prediction intervals for *θ*_*i*_. The first justification is likely to appeal most to those who prefer to interpret ${\hat{\theta }}_{i}$ as the empirical Bayes estimate and the second to those who prefer to interpret it as the BLUP.

One salient point is that, by virtue of ${\hat{\theta }}_{i}$ being the BLUP, Var(${\hat{\theta }}_{i}-\theta_{i})$ is less than Var($Y_{i}-\theta_{i})=\sigma_{i}^{2}$. This is most easily seen by considering the ratio of Equation (10) and $\sigma_{i}^{2}$. If there is one study then $1/w=\sigma_{i}^{2}+\tau^{2}$ and this ratio is one. However if there are two or more studies then $1/w<\sigma_{i}^{2}+\tau^{2}$, and hence the ratio is also less than one, so that Equation (10) is less than $\sigma_{i}^{2}$ as required. It is in this sense that ${\hat{\theta }}_{i}$ is the 'better' estimate of *θ*_*i*_ under the random‐effects model.

### 3.3 Quan et al. formula

Quan et al.^24^ develop methodology in the context of multicenter trials but their approach is essentially a conventional random‐effects meta‐analysis. Here each center provides an effect size estimate of a raw mean difference *Y*_*i*_ and the random‐effects model for meta‐analysis is applied to the resulting estimates; Quan et al.^24^ estimate the within‐study variances as $\sigma_{i}^{2}=2\sigma^{2}/N_{i}$ where *σ* ^2^ is the individual level variance and *N*_*i*_ is the number of patients in each of two treatments arms in the *i*th study. Quan et al.^24^ use the DerSimonian and Laird estimator of *τ* ^2^ and also present the empirical Bayes estimate in their Equation (10). Note that Quan et al.^24^ used the raw mean difference as effect size measure, and therefore their $\sigma_{i}^{2}$ has this particular form. However, their result may be used more generally where $\sigma_{i}^{2}$ is the within‐study variance of any other effect size measure.

Quan et al.^24^ correctly state that (taking ${\hat{\tau }}^{2}$ to be *τ* ^2^) ${\hat{\theta }}_{i}$ is unbiased for *θ* so that $E\left[{\hat{\theta }}_{i}\right]=\theta$. They also correctly state the variance of ${\hat{\theta }}_{i}$ as being given by Equation (13) where $\sigma_{i}^{2}=2\sigma^{2}/N_{i}$. However, we would like to take this opportunity to alert readers to an important subtlety. If this variance is used, then the pivot for making inferences is

(16) $$\left({\hat{\theta }}_{i}-\theta \right)∼N\left(0,\mathrm{Var}\left({\hat{\theta }}_{i}\right)\right).$$

Note that this is different from the pivot used in pivot (11) that makes inferences for *θ*_*i*_; pivot (16) instead makes inferences for *θ*. Hence, using Equation (13) as the variance of $\hat{\theta_{i}}$ is ill‐advised and suboptimal. We suggest that inferences for *θ* would be better served using $\hat{\theta }$ rather than ${\hat{\theta }}_{i}$.

#### 3.3.1 Further consequences of the Quan et al. formula

To better understand the consequences of using the Quan et al. formula in Equation (13), let us simplify matters and assume that the number of studies is large, and so *w* is large. In this situation, the second term of Equation (10) is negligible in relation to the first and (10), and so the Raudenbush formula is approximately

(17) $$\mathrm{Var}\left(\theta_{i}|y,\tau^{2}\right)=V_{i}\approx \frac{\sigma_{i}^{2}\tau^{2}}{\sigma_{i}^{2}+\tau^{2}}.$$

Furthermore in this situation $\mathrm{Var}\left({\hat{\theta }}_{i}\right)$ is approximately

(18) $$\mathrm{Var}\left({\hat{\theta }}_{i}\right)\approx \frac{\tau^{4}}{\sigma_{i}^{2}+\tau^{2}}.$$

Comparing Equations (17) and (18), we can see that the Raudenbush and Quan et al. formulae are asymptotically identical if $\tau^{2}=\sigma_{i}^{2}$, which means that the within‐study and between‐study variances are the same. Hence, these formulae can be expected to give similar numerical answers in many applications. More generally, the implications of using the Raudenbush and Quan et al. formulae in large samples can be deduced from Equations (17) and (18).

It is straightforward to show that Equation (17) is increasing in $\sigma_{i}^{2}$. Hence, small studies (large $\sigma_{i}^{2}$) provide large values of (17) and so wide intervals for *θ*_*i*_ when using this variance. This makes intuitive sense: small studies provide less information, and these result in less informative inference for *θ*_*i*_. It is also straightforward to show that Equation (18) is decreasing in $\sigma_{i}^{2}$. Hence, small studies provide small values of Equation (18), and these result in narrow intervals. This may seem counterintuitive, but recall that the Quan et al. formula is suitable for calculating intervals for *θ* while being suboptimal for calculating intervals for *θ*_*i*_.

### 3.4 Recommendation

We recommend using Raudenbush formula in Equation (10) when computing prediction intervals for the study specific true effects *θ*_*i*_. It is clearly an improvement on the crude formula and, unlike the Quan et al. formula, is entirely suited to this purpose. We interpret this formula as giving an approximate posterior variance of ${\hat{\theta }}_{i}$ when interpreting this as the empirical Bayes estimate, and as an approximate variance of the estimation error $\left({\hat{\theta }}_{i}-\theta_{i}\right)$ when interpreting ${\hat{\theta }}_{i}$ as the BLUP.

## 4 EXAMPLES

In this section, we calculate the empirical Bayes estimates and corresponding study specific 95% prediction intervals for primary studies in two example meta‐analyses. We graphically present the results by adding the empirical Bayes estimates and prediction intervals to forest plots.^17^ The examples were selected to represent meta‐analyses with small and large between‐study variances. R^36^ and the R package metafor^35^ were used for conducting the meta‐analyses and creating the forest plots. R code of these analyses are available via https://osf.io/sz6ka/.

Our hope is that these two contrasting examples will illustrate the practical utility of the methods that provide our focus, and they will serve to encourage the reader to apply them to their own examples.

### 4.1 Characteristics of the examples

#### 4.1.1 Example one: very little between‐study heterogeneity

The first example is a meta‐analysis by Ho and Lee^37^ on the efficacy of eye movement desensitization (EMDR) and reprocessing versus exposure based cognitive behaviour therapy (CBT) to treat post‐traumatic stress disorder (PTSD). This meta‐analysis contains 10 Hedges' *g* standardized mean differences and a positive effect size indicates that EMDR was more efficacious than CBT. Fitting the random‐effects model to these data with the restricted maximum likelihood (REML) estimator for the between‐study variance yielded $\hat{\theta }=0.249$ (95% CI [−0.003;0.502]), ${\hat{\tau }}^{2}=0.004$, and ratio of the between‐study variance to total variance equal to *I* ^2^ = 2.2*%*.^38^

#### 4.1.2 Example two: substantial between‐study heterogeneity

The second example is a meta‐analysis on the difference in iron blood levels between patients with Alzheimer's disease and elderly controls.^39^ This meta‐analysis consists of five Hedges' *g* standardized mean differences where larger effect sizes indicate higher iron levels for patients with Alzheimer's disease. Results of fitting the random‐effects model to these data were $\hat{\theta }=-0.177$ (95% CI [−0.486;0.132]), ${\hat{\tau }}^{2}=0.063$, and *I* ^2^ = 51.6*%*.

### 4.2 Results

**FIGURE 1:**
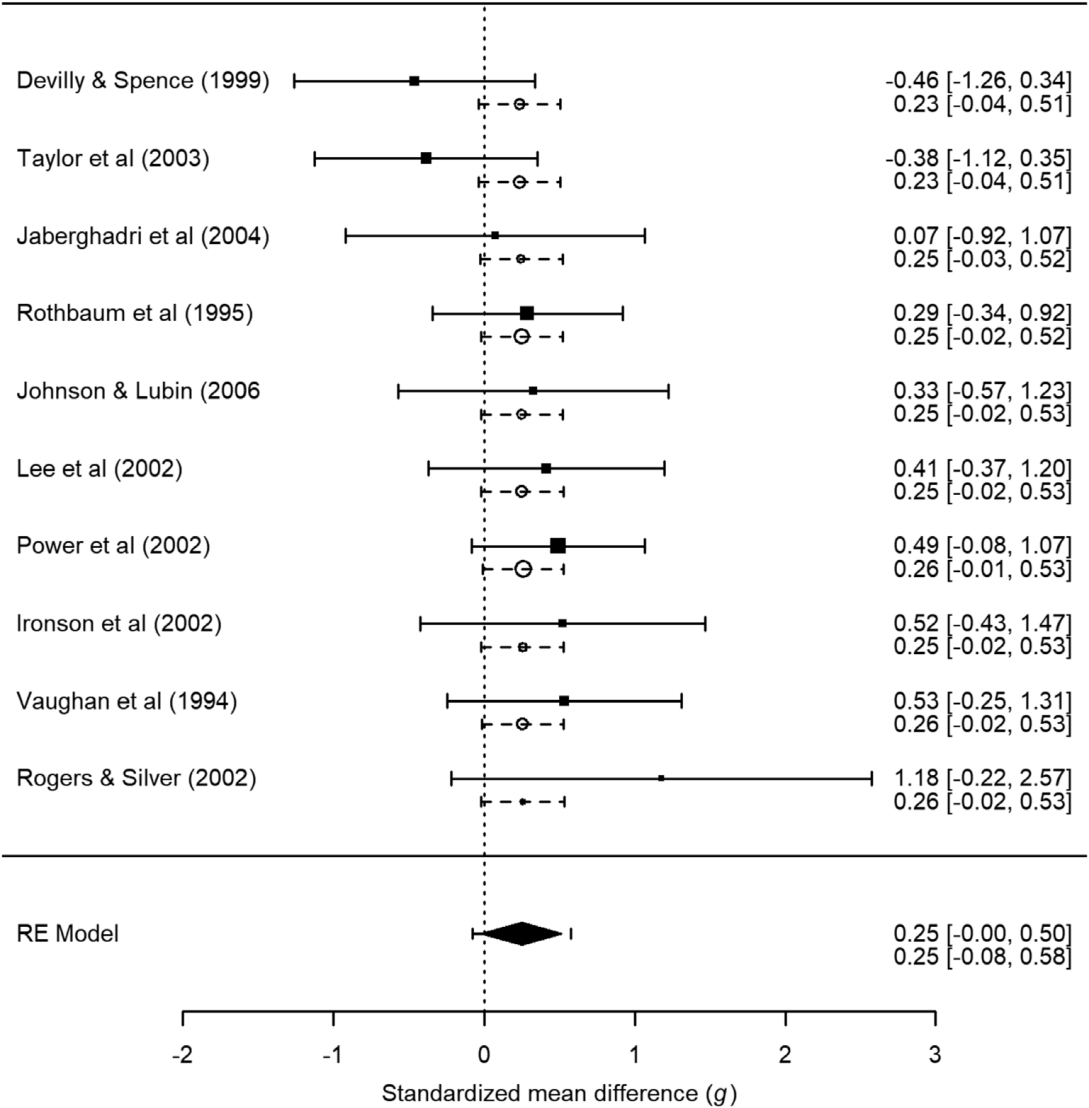
Forest plot including empirical Bayes estimates (circles) and study specific 95% prediction intervals based on the Raudenbush formula in Equation (10) (dashed lines) for the meta‐analysis by Ho and Lee^37^ on the efficacy of eye movement desensitization and reprocessing versus exposure based cognitive behaviour therapy to treat post‐traumatic stress disorder. RE Model refers to random‐effects model

**FIGURE 2:**
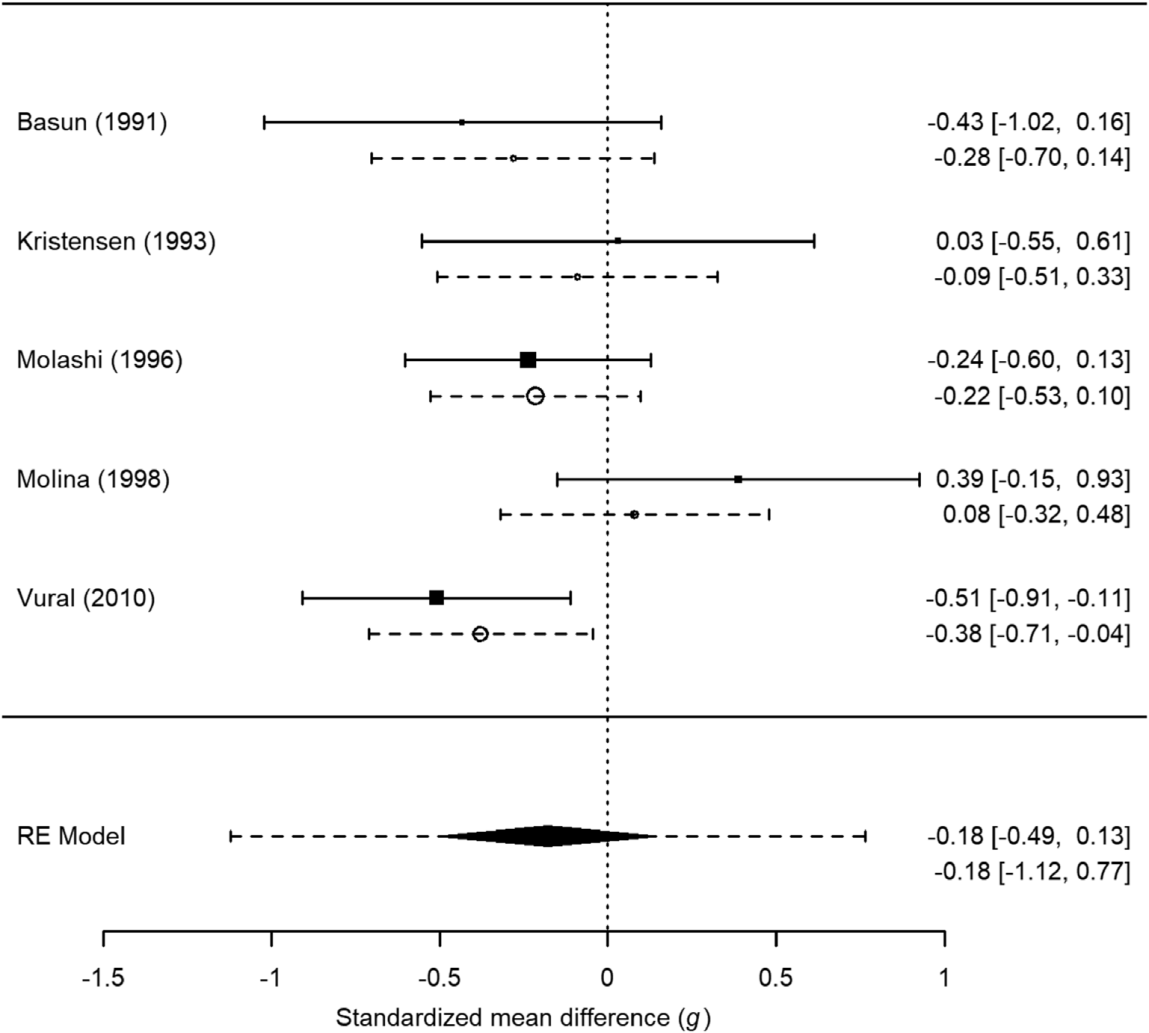
Forest plot including empirical Bayes estimates (circles) and study specific 95% prediction intervals based on the Raudenbush formula in Equation (10) (dashed lines) for the meta‐analysis by Lopes da Silva et al.^39^ on the difference in iron blood levels between patients with Alzheimer's disease and elderly controls. RE Model refers to random‐effects model

Figures 1 and 2 show the forest plots of the meta‐analysis on the efficacy of the PTSD treatments and the difference in iron blood levels between patients with Alzheimer's disease and elderly controls, respectively. The results of fitting the random‐effects model are illustrated by the diamond at the bottom of the plots. The diamond's midpoint refers to $\hat{\theta }$ and its width indicates the lower and upper bound of the 95% confidence interval. The dashed lines connected to the diamond illustrate the prediction interval for the true effect in a new study that is computed with $\hat{\theta }\pm t_{n-2;0.975}\sqrt{1/\sum {\hat{w}}_{i}+{\hat{\tau }}^{2}}$ where *t*_*n* − 2;0.975_ is the 0.975 quantile of the Student's *t*‐distribution with *n* − 2 degrees of freedom.^5^, ^6^ The observed effect sizes of the primary studies (i.e., *Y*_*i*_) and the corresponding 95% confidence intervals are displayed by squares and solid black lines, respectively. Empirical Bayes estimates (i.e., $\hat{\theta_{i}}$) are presented by circles and the study specific 95% prediction intervals based on the Raudenbush formula in (10) are illustrated by dashed lines. The sizes of the squares and circles reflect the precision of a primary study with larger squares and circles referring to primary studies that are more precise.

### 4.3 Comparing the results for the two examples

The nature of the shrinkage behaviour of empirical Bayes estimates is illustrated by our two contrasting examples. The empirical Bayes estimates in Figure 1, for our first example with very little between‐study heterogeneity are substantially shrunken to $\hat{\theta }$. This is a direct consequence of Equation (4) when ${\hat{\tau }}^{2}$ is small. This example illustrates the considerable impact that additional information, that is available under the random‐effects model but not conventionally used, can have on the estimates of the study specific effects under these circumstances. Much more accurate predictions of the study specific true effects are also possible when using prediction, rather than the confidence, intervals.

Two other interesting observations can also be made based on Figure 1. First, the empirical Bayes estimates of the first two studies (i.e., Devilly & Spence and Taylor et al.) are of different sign to the observed effect size estimates due to the shrinkage, which might be seen as controversial. Second, the prediction interval for the true effect in a new study at the bottom of Figure 1 is noticeably wider than all study specific prediction intervals and the confidence interval for the pooled effect under the random‐effects model. This is despite the fact that the between‐study variance is very close to zero (${\hat{\tau }}^{2}$ = 0.004). All three types of intervals might be expected to coincide if ${\hat{\tau }}^{2}$ = 0 (see the discussion in the last paragraph of section 3.2). However, the prediction interval for the true effect in a new study is noticeably wider for two reasons: (1) ${\hat{\tau }}^{2}$ is slightly positive, and (2) we adopted the convention of using the Student's *t*‐distribution with *n* − 2 degrees of freedom when computing it.^5^, ^6^

Shrinkage of the empirical Bayes estimates is also present in the second example, with substantial between‐study heterogeneity, shown in Figure 2. However, the shrinkage is substantially smaller and the width of the study specific prediction intervals are comparable to the corresponding confidence intervals. This difference in shrinkage behaviour in these two examples can be explained by the smaller heterogeneity in the first compared to the second example (*I* ^2^ = 2.2*%* vs. 51.6%). This is because as *τ* ^2^ → ∞, Equation (4) tends towards *Y*_*i*_ and (for example, using L'Hopital's rule) Equation (10) tends towards $\sigma_{i}^{2}$ (the second term in Equation 4 tends to zero as *τ* ^2^ → ∞). Hence there is less information gain by using empirical Bayes estimates and study specific prediction intervals in heterogeneous datasets, but our examples illustrate what is possible in this regard for real examples.

These examples illustrate the usefulness of embellishing forest plots with prediction intervals. We, however, recommend meta‐analysts to always present confidence and prediction intervals for the study‐specific effects as well as for the average effect as these all add insight to the meta‐analysis.

## 5 ORIGINAL RESEARCH: SIMULATION STUDY

Most of this tutorial is devoted to distilling the statistical theory, illustrating its use, and making recommendations for best practice. However in this section we will enhance this tutorial by conducting a simulation study. This is important because the random‐effects model requires strong assumptions that are usually not tenable in practice. For example, uncertainty in the estimation of *τ* ^2^ is usually ignored and the sampling distribution of a study's effect size is assumed to follow a normal distribution. We assess the robustness of study specific 95% prediction intervals to violations of these assumptions in two simulation studies. Study specific 95% prediction intervals were computed using the required variance formula and the critical value of 1.96 from the standard normal distribution. We mainly used the Raudenbush formula in (10) for computing the study specific prediction intervals, but also used the Quan formula in Equation (13) to show its impact. An overview of the conditions included in the simulation studies is presented in Table 1.

**TABLE 1:** Overview of conditions in the two simulation studies. *μ* is the average true effect, *n* is the number of studies in a meta‐analysis, *I* ^2^ is the *I* ^2^‐statistic to quantify the between‐study heterogeneity, $\sigma_{i}^{2}$ is the true sampling variance of the *i*th study, $\pi_{i}^{c}$ is the true probability of the outcome of interest in the control group of the *i*th study, $n_{i}^{c}$ and $n_{i}^{e}$ are the sample sizes in the control and experimental group of the *i*th study. 10,000 simulated datasets were produced under each condition

	Simulation study	
	Normally distributed	Binary
*μ*	0	0
*n*	5; 20; 80	5; 20; 80
*I* ^2^	0; 0.1; 0.25; 0.5; 0.75; 0.9	0; 0.1; 0.25; 0.5; 0.75; 0.9
$\sigma_{i}^{2}$	unif(0.05, 1)	–
$\pi_{i}^{c}$	–	0.1; 0.5
$n_{i}^{c}=n_{i}^{e}$	–	50; 200

### 5.1 Methodology for simulating data

We performed two types of simulation studies. In the first we assume that the random‐effects model is true, but allow for the fact that *τ* ^2^ is unknown. In the second we simulate comparative binary outcome data and use normal approximations so that the random‐effects model is merely an approximation. Our expectation was that our methods would perform better in the first simulation study than the second.

Both simulation studies were programmed in R^36^ and the R package metafor^35^ was used for computing the empirical Bayes estimates and their study specific 95% prediction intervals. Results of both simulation studies were based on 10,000 replications per condition. R code of the simulation studies is available at https://osf.io/4uq93/ and all results of the simulation studies are in the web Supplementary materials.

#### 5.1.1 Simulation study 1: normally distributed outcome data

For each simulated meta‐analysis, data were generated by first sampling *i* = 1, …, *n* true effect sizes from a normal distribution, *θ*_*i*_ ∼ *N*(*μ*, *τ* ^2^). A sampling variance $\sigma_{i}^{2}$ was drawn from a uniform distribution with 0.05 and 1 as lower and upper bound, respectively. This is merely intended as a simple and transparent way to draw sampling variances; uniform distributions have previously been used for this purpose.^40^ Subsequently, the observed effect sizes *Y*_*i*_ were sampled from $N\left(\theta_{i},\sigma_{i}^{2}\right)$.

The average effect size in the population was fixed to *μ* = 0, because for normally distributed outcome data this parameter only changes the location of the observed effect size estimates without otherwise affecting the statistical properties of the study specific 95% prediction intervals. Three levels for the number of effect sizes in a meta‐analysis were selected, *n* = 5, 20 and 80. These levels resemble the number of effect sizes of meta‐analyses published in the Cochrane Database of Systematic Reviews, because the median number of effect sizes in these meta‐analyses is three and the 99*th* percentile is 28 effect sizes.^41^, ^42^ The condition with 80 effect sizes in a meta‐analysis was added to assess the statistical properties for a large number of effect sizes. Values for *τ* ^2^ were selected to correspond to *I* ^2^‐statistics equal to 0, 0.1, 0.25, 0.5, 0.75 and 0.9, where *I* ^2^ = *τ* ^2^/(*s* ^2^ + *τ* ^2^) and *s* ^2^ is the 'typical' within‐study variance. In order to be able to compute these *τ* ^2^ values from the required *I* ^2^, we computed *s* ^2^ = 0.317 to 3 decimal places, given that the $\sigma_{i}^{2}$ are sampled from a uniform distribution with 0.05 as lower bound and 1 as upper bound, using the approach described by Jackson and Bowden.^43^

With three different numbers of studies *n* = 5, 20, 80, and six different values of *I* ^2^ = 0, 0.25, 0.5, 0.75, 0.9 corresponding to ${\hat{\tau }}^{2}=0,0.035,0.106,0.317,0.951,2.854$, this simulation study examines 18 different conditions.

#### 5.1.2 Simulation study 2: comparative binary outcome data

For each *i* = 1, …, *n* primary study in a meta‐analysis, we first sampled a true effect size *θ*_*i*_ from a normal distribution with mean *μ* and variance *τ* ^2^ as in the first simulation study. Based on this *θ*_*i*_, we created for each *i*th primary study a 2 × 2 frequency table by first generating the number of cases with the outcome of interest in the control group, $x_{i}^{c}∼B\left(n_{i}^{c},\pi_{i}^{c}\right)$ where *B* refers to the binomial distribution, $n_{i}^{c}$ to the sample size in the control group, and $\pi_{i}^{c}$ to the true probability of the outcome of interest in the control group. The true probability of the outcome of interest in the experimental group is a function of *θ*_*i*_ and $\pi_{i}^{c}$ and was computed with $\pi_{i}^{e}=\pi_{i}^{c}\times \exp\left(\theta_{i}\right)/\left[1-\pi_{i}^{c}+\pi_{i}^{c}\times \exp\left(\theta_{i}\right)\right]$. The number of cases with the outcome of interest in the experimental group ($x_{i}^{e}$) was then sampled from $B\left(n_{i}^{e},\pi_{i}^{e}\right)$ where $n_{i}^{e}$ is the total number of cases in the experimental group. Before we computed the log odds ratio and its sampling variance, we added 0.5 to all cells of the 2 × 2 frequency table to decrease bias in the estimator of the log odds ratio and enable the computation of the sampling variance in case of zero cells.^44^ The log odds ratio was computed with

$$Y_{i}=\log\left(\frac{x_{i}^{e}}{n_{i}^{e}-x_{i}^{e}}/\frac{x_{i}^{c}}{n_{i}^{c}-x_{i}^{c}}\right)$$

and its corresponding sampling variance with

(19) $${\hat{\sigma }}_{i}^{2}=\frac{1}{x_{i}^{e}}+\frac{1}{n_{i}^{e}-x_{i}^{e}}+\frac{1}{x_{i}^{c}}+\frac{1}{n_{i}^{c}-x_{i}^{c}}.$$

The average log odds ratio in the population was fixed to *μ* = 0, as in the first simulation study. Unlike in the first simulation study, the choice of *μ* is not immaterial anymore, but we expected this to have a relatively minor impact and so instead varied other factors that we deemed as more important. This is to keep the number of conditions examined manageable. The true probability of the outcome of interest in the control group was set equal to either $\pi_{i}^{c}=0.1$ or 0.5 in all studies. The number of patients in the control group and treatment group was set to either $n_{i}^{c}=n_{i}^{e}=50$ or 200. As in the first simulation study, the number of effect sizes in the meta‐analysis was set equal to *n* = 5, 20 or 80.

Similar to the first simulation study, values for *τ* ^2^ were selected to represent *I* ^2^‐statistics of 0, 0.1, 0.25, 0.5, 0.75, 0.9. However, the typical within‐study sampling variance, *s* ^2^, in the identity *I* ^2^ = *τ* ^2^/(*s* ^2^ + *τ* ^2^), cannot be computed in the same way as in simulation study 1, because we no longer have a distribution of within‐study variances immediately available to us. We therefore computed approximate typical within‐study variances using the standard formula for a log odds, $s^{2}=1/\left(n_{i}^{c}\pi_{i}^{c}\left(1-\pi_{i}^{c}\right)\right)+1/\left(n_{i}^{e}\pi_{i}^{e}\left(1-\pi_{i}^{e}\right)\right)$, where we further approximated (because we set *μ* = 0) $\pi_{i}^{e}=\pi_{i}^{c}$. This approach is especially simple and transparent, but gives very similar typical within‐study variances to a simulation based approach that was also considered. In this alternative approach, studies were simulated assuming $\pi_{i}^{e}=\pi_{i}^{c}$, within‐study variances were computed using Equation (19) and then the typical within‐study variance was computed using Equation (9) of Higgins and Thompson.^38^

With three different numbers of studies *n* = 5, 20, 80, six different values of *I* ^2^ = 0, 0.1, 0.25, 0.5, 0.75, 0.9, and four combinations of $n_{i}^{c}=n_{i}^{e}=50$ or 200 and $\pi_{i}^{c}=0.1$ or 0.5, this simulation study examines 72 different conditions.

### 5.2 Assessing the performance of study specific prediction intervals

Study specific 95% prediction intervals were computed using the Raudenbush formula in Equation (10). Some results were also produced using the Quan et al. formula in Equation (13) to assess the impact of using it. The true *τ* ^2^ was initially used in both variance formulae to examine whether the coverage probabilities of the prediction intervals were equal to the nominal coverage rate, as expected according to theory in simulation study 1. However our primary interest is in the statistical properties of the study specific prediction intervals if *τ* ^2^ was estimated. We included the widely used DerSimonian and Laird^2^ estimator as well as the currently recommended^7^, ^8^ REML^18^ and Paule‐Mandel^45^ estimators for estimating *τ* ^2^. Note that the Paule‐Mandel estimator is equivalent to an estimator for *τ* ^2^ that has been developed in the context of empirical Bayes estimation.^46^, ^47^, ^48^

The main outcome of interest in both simulation studies was the coverage probability of the study specific 95% prediction intervals. For each generated meta‐analysis, we assessed whether each *θ*_*i*_ fell within the bounds of its prediction interval and computed the average coverage probability based on all studies in a meta‐analysis. These average coverage probabilities per meta‐analysis were then averaged over all simulated datasets.

#### 5.2.1 Additional assessments

The coverage probabilities of the study specific prediction intervals were also computed for only the largest *θ*_*i*_ in a meta‐analysis (in case of *τ* ^2^ = 0 coverage probability of the first *θ*_*i*_ was assessed). This was to assess their performance for the most outlying trial. For the first simulation study, we also computed the coverage probability of the study with the largest $\sigma_{i}^{2}$, that is, the 'smallest' study. The performance for the smallest study was examined as it was thought that prediction intervals might then perform poorly, because small studies' results are shrunk the most to the pooled effect. Another outcome of interest in both simulation studies was the average width of the study specific prediction intervals.

Study specific prediction intervals were also computed in combination with the Knapp–Hartung adjustment^49^, ^50^, ^51^, ^52^ if an estimate of *τ* ^2^ was used. The Knapp–Hartung adjustment takes uncertainty in estimates of the variance components into account by scaling the variance of $\hat{\theta }$ (i.e., 1/*w*) and by using the *t*‐distribution with *n* − 1 degrees of freedom as pivot.^53^ The Raudenbush formula in Equation (10) also contains that variance of $\hat{\theta }$, 1/*w*, and this quantity is scaled when computing study specific prediction intervals in combination with the Knapp–Hartung adjustment. Simulation studies^54^, ^55^, ^56^ have shown that the Knapp–Hartung adjustment yields more accurate coverage probabilities of 95% confidence intervals than conventional random‐effects meta‐analysis. However, it is unknown whether prediction intervals become more accurate if the adjustment is used. The results for all of these additional assessments are contained in the web Supplementary materials.

### 5.3 Properties of prediction intervals using the recommended Raudenbush variance formula

**FIGURE 3:**
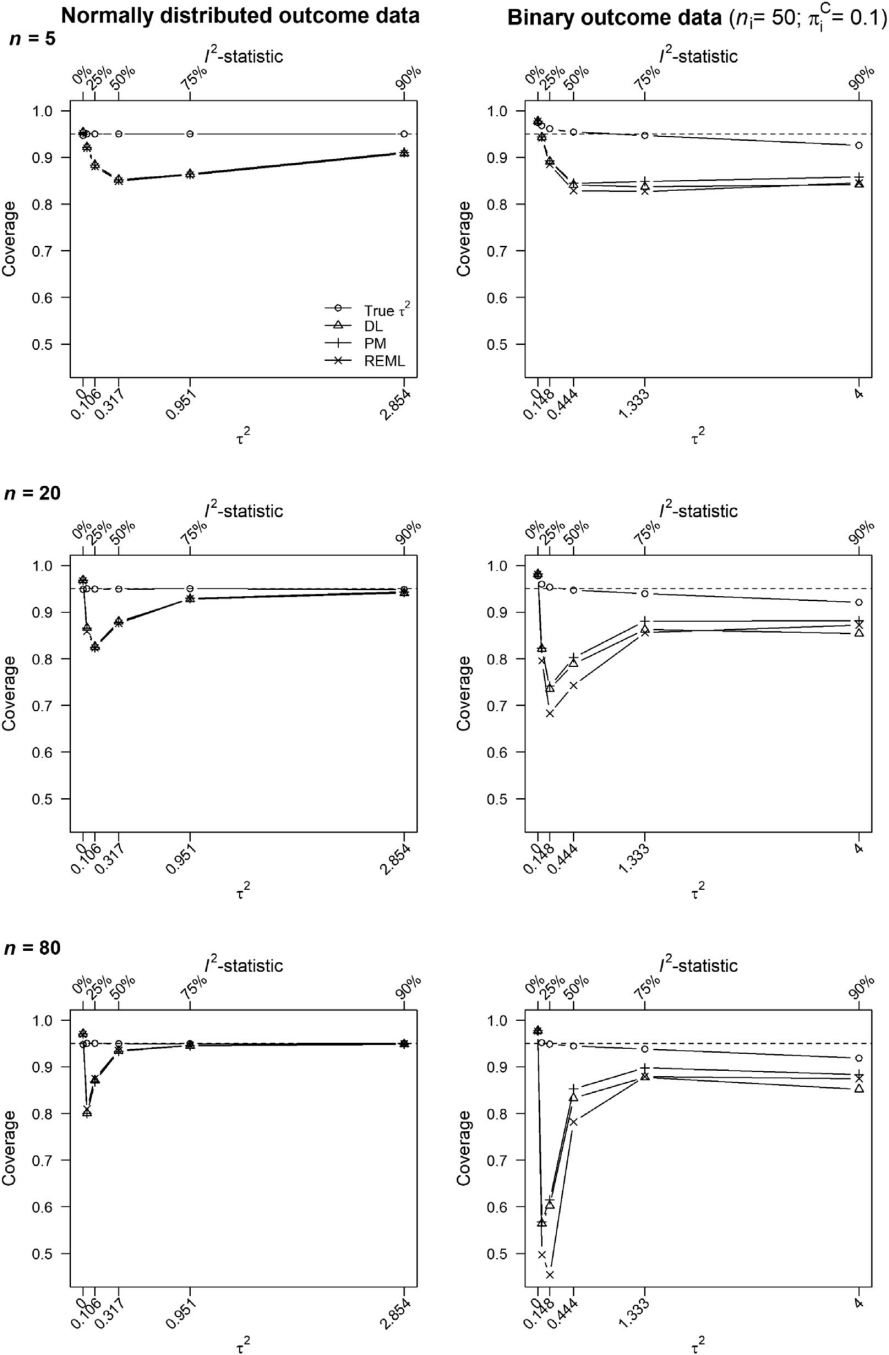
Coverage probability of study specific 95% prediction interval for normally distributed outcome data (left column) and binary outcome data (right column) as function of heterogeneity in true effect size (*x*‐axis). The Raudenbush formula in Equation (10) was used for estimating the variance of the empirical Bayes estimates. Circles denote coverage probability when the true *τ* ^2^ was used for computing the 95% prediction interval and triangles, plus signs, and crosses denote that *τ* ^2^ was estimated using the DerSimonian and Laird,^2^ Paule‐Mandel,^45^ and restricted maximum likelihood (REML)^18^ estimator. The rows present coverage probabilities in meta‐analyses based on *n* = 5, 20, and 80 studies

Figure 3 shows the coverage probabilities where coverage probabilities using the true *τ* ^2^ are denoted by circles. Triangles, plus signs, and crosses denote coverage probabilities when *τ* ^2^ was estimated using DerSimonian and Laird, Paule‐Mandel, and restricted maximum likelihood, respectively. In the first simulation study, with normally distributed outcome data (left column), coverage probabilities were equal to the nominal coverage rate if the true *τ* ^2^ was used. This was as expected, because there is no uncertainty in estimation of *τ* ^2^ and all the assumptions made when deriving the prediction intervals are true. If *τ* ^2^ was estimated, as will be the case in practice, the coverage probability based on all three estimators was highly comparable. It was slightly above the nominal coverage rate if *I* ^2^ = 0*%* and was too low if *I* ^2^ > 0*%*.

We present the coverage probabilities of study specific 95% prediction intervals for the conditions *n*_*i*_ = 50 and $\pi_{i}^{C}=0.1$ for the second simulation study (binary outcome data) in the right hand column of Figure 3. These conditions were selected because this was the worst‐case scenario (smallest studies and event probabilities) where coverage probabilities deviated the most from the nominal coverage rate. The results for the other three combinations of *n*_*i*_ and $\pi_{i}^{C}$ are shown in the web Supplementary materials. The general pattern of results are similar in the two columns of Figure 3, but the deviations from the nominal coverage probability of 95% is greatest for the second simulation study, as expected. Study specific prediction intervals based on the REML estimator for the between‐study variance deviated the most from the nominal coverage probability, because it was most negatively biased resulting in, on average, the narrowest intervals. As also expected, deviations from the nominal coverage rate decreased when $n_{i}^{c}=n_{i}^{e}=200$ compared to 50 in the second simulation study, and also when the event was more common, $\pi_{i}^{c}=0.5$ (see web Supplementary materials). These observations can be explained because the normal approximations made by the random‐effects model are then more accurate.

#### 5.3.1 A defect in the study specific prediction intervals

Two recent studies^57^, ^58^ examined the coverage probability of prediction intervals for the true effect in a new study. Both studies observe low coverage probabilities if *τ* ^2^ is small but non‐negligible. We observe the same defect for study specific prediction intervals when *τ* ^2^ is estimated (Figure 3). This suggests that we need to treat the prediction intervals for our first example in Figure 1 with caution, because here ${\hat{\tau }}^{2}$ was small but non‐negligible raising concerns that their actual coverage probability might substantially deviate from the nominal coverage rate of 95%.

To understand why these defects occur, imagine a meta‐analysis where *τ* ^2^ is small compared to $\sigma_{i}^{2}$ (but non‐negligible) and (to simplify matters) the number of studies is large. In such a meta‐analysis, the magnitude of the variance in Equation (10) is mainly determined by the first term, because 1/*w* in the second term is small. By dividing the numerator and the denominator of the first term by $\sigma_{i}^{2}$, we can see that this variance is approximately equal to *τ* ^2^. The assumed variance used in the calculation of the prediction interval for the true effect of a new study is 1/*w* + *τ* ^2^, and this is also approximately equal to *τ* ^2^. This shows that the variance used in the computation of both types of prediction intervals is approximately *τ* ^2^ in this type of situation. The uncertainty in this parameter is ignored, which largely explains the under coverage of both types of prediction intervals. Furthermore, *τ* ^2^ is often estimated as zero for small but non‐negligible *τ* ^2^ and this results in too narrow intervals for both types of prediction intervals. Small *τ* ^2^ and large *n* therefore appeared to be a 'perfect storm' for both types of prediction intervals to perform poorly. For larger values of *τ* ^2^, the performance of the prediction intervals improved as *n* increased, as one might expect. The only scenario in our simulation study where the prediction intervals yielded over coverage were for the conditions where *τ* ^2^ = 0, again as one might expect.

#### 5.3.2 Additional assessments

See the web Supplementary materials for all results concerning the additional assessments. We did not interpret the average width of the study specific prediction intervals, because a prerequisite for interpreting the width is that the coverage probability is close to the nominal coverage rate, which was not the case. The coverage probability of study specific prediction intervals were generally slightly closer to the nominal coverage rate if the Knapp–Hartung adjustment was used, where this improvement was particularly notable for *n* = 5. More notable under coverage of study specific prediction intervals was observed for the prediction intervals with the largest *θ*_*i*_. This is an important observation, indicating that the study specific prediction intervals that we computed do not perform well if we condition on estimating the largest *θ*_*i*_. However, this appeared to be a less serious issue in situations where the between‐study variance was either very small or considerable. These observations can be explained because if *τ* ^2^ was small then there was very little variation in the *θ*_*i*_, and if *τ* ^2^ was large then there was very little shrinkage so that study specific prediction intervals were similar to conventional confidence intervals. The coverage probabilities of prediction intervals for the study with the smallest study in a meta‐analysis were comparable to the average coverage probability of the prediction intervals of all studies in a meta‐analysis.

### 5.4 Properties of prediction intervals using the Quan et al. variance formula

**FIGURE 4:**
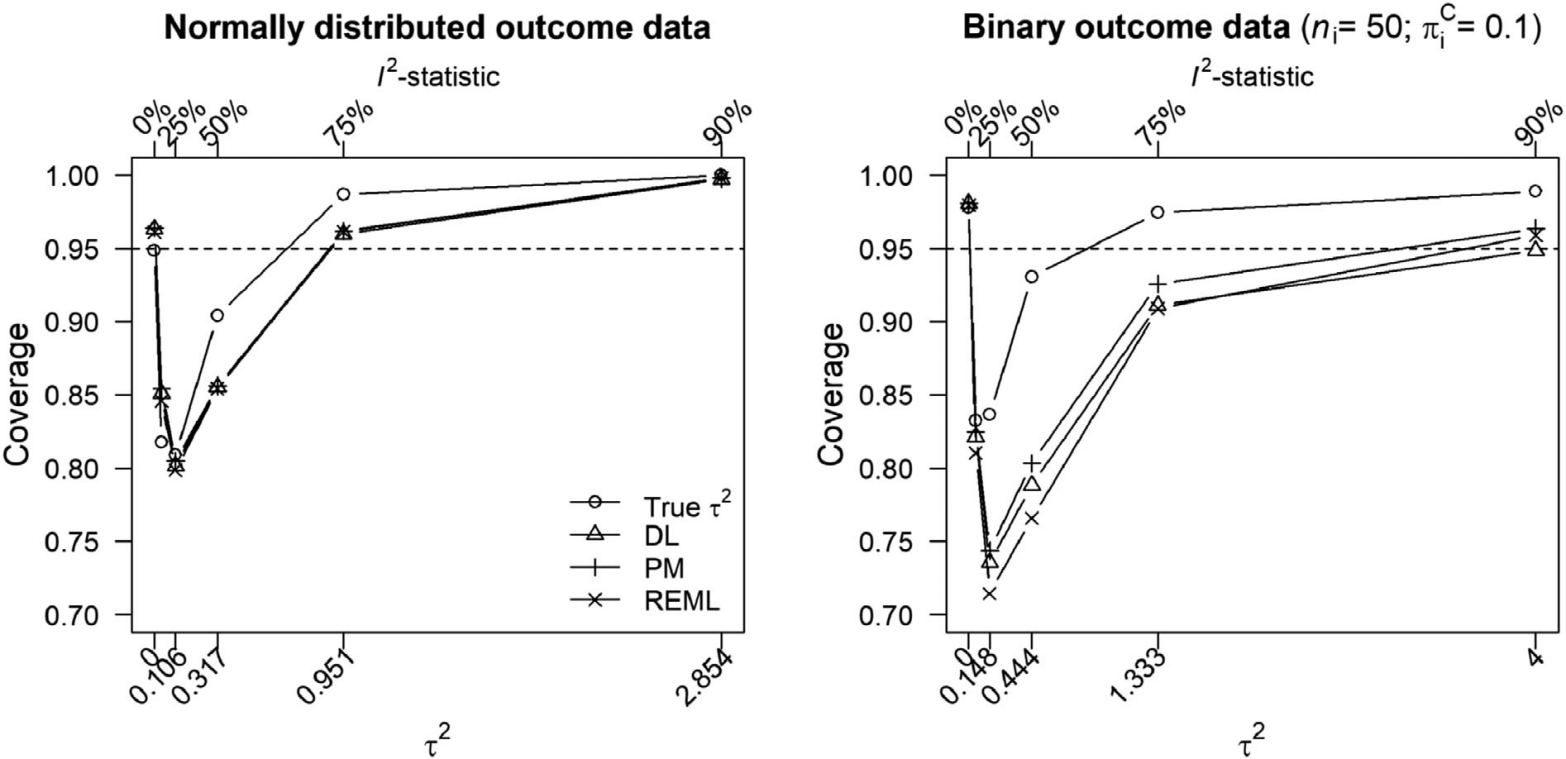
Coverage probability of the study specific 95% prediction interval for normally distributed outcome data (left panel) and binary outcome data (right panel) as function of heterogeneity in true effect size (*x*‐axis). The Quan et al. formula was used for estimating the variance of the empirical Bayes estimates. Circles denote coverage probability when the true *τ* ^2^ was used for computing the 95% prediction interval and triangles, plus signs, and crosses denote that *τ* ^2^ was estimated using the DerSimonian and Laird,^2^ Paule‐Mandel,^45^ and restricted maximum likelihood (REML)^18^ estimator. Both figures show the results of *n* = 20 studies in a meta‐analysis

As explained in Section 5.2, some results were produced using the Quan et al. variance formula. For the first simulation study, with normally distributed outcome data, results for *n* = 20 were produced. For the second simulation study, with binary outcome data, results for *n* = 20, $n_{i}^{c}=n_{i}^{e}=50$, and $\pi_{i}^{c}=0.1$ were produced. Figure 4 shows the resulting coverage probabilities of 95% prediction intervals, for *θ*_*i*_, using the Quan et al. formula. As expected from the discussion in Section 3.3.1, the Quan et al. variance formula provided coverage probabilities that were too large when *τ* ^2^ was large, and coverage probabilities that were too small when *τ* ^2^ was small. This under coverage when *τ* ^2^ was small increased as a function of *n* (see web Supplementary materials). From Equations (17) and (18), one might expect the Quan et al. formula to produce the correct coverage probability when *I* ^2^ = 0.5, the random‐effects model is true and the true value of *τ* ^2^ is used. This was approximately the case in the left hand side of Figure 4. However, the majority of simulated within‐study variances, that were uniformly distributed in the interval 0.05 to 1, were greater than the typical within‐study variance of 0.317. Hence, *τ* ^2^ was then less than the majority of simulated within‐study variances when *I* ^2^ = 0.5; from Equations (17) and (18) we then expect some under coverage, as was the case in Figure 4.

### 5.5 Overall conclusions

In general, 95% study specific prediction intervals using the Raudenbush variance formula have performed well. However, they have the defect that they provide under coverage in situations where *τ* ^2^ is small but non‐negligible. This is mainly caused by estimation of *τ* ^2^, because coverage probabilities are close to the nominal coverage rate if the true *τ* ^2^ rather than an estimate is used for creating the study specific prediction intervals. As expected, study specific prediction intervals perform worse in situations where the normal approximations made by the random‐effects model are less accurate. The Quan et al. formula does not reliably provide the correct coverage probability for *θ*_*i*_, even in situations where the random‐effects model was true and *τ* ^2^ was known.

## 6 DISCUSSION

Estimates of study specific effects are often of interest. For example, a particular study may be conducted in a population of particular interest. We recommend using the empirical Bayes estimates, or equivalently the BLUPs, with study specific prediction intervals, to make the necessary statistical inferences. We suggest using these quantities in addition to the usual study specific estimates and to confidence intervals shown on forest plots. In hindsight, it may even seem strange to suggest presenting prediction intervals for the true effect in a new study in forest plots, as sometimes advocated,^6^, ^59^ but not suggesting to also include study specific prediction intervals.^5^, ^6^, ^30^

Our tutorial contributes to the existing literature in three ways. First, we have coalesced and elucidated the literature on empirical Bayes estimates, BLUPs, and study specific prediction intervals, and have examined three different variance formulae for computing these prediction intervals. This revealed that the Quan et al. formula makes inferences for the average effect, rather than study specific effects. Hence, we recommend researchers who want to compute study specific prediction intervals to use the Raudenbush rather than the Quan et al. formula, because the latter is based on an inappropriate pivot. The second contribution is illustrating how forest plots can be embellished by adding empirical Bayes estimates or BLUPs together with their corresponding prediction intervals. We have illustrated this by creating forest plots for meta‐analyses on the efficacy of treatments for PTSD and iron blood levels of patients with Alzheimer's disease. Embellishing forest plots with empirical Bayes estimators or BLUPs with prediction intervals has been suggested before,^21^, ^22^, ^23^ but this type of plot is not yet routinely used. One concrete recommendation is to routinely embellish forest plots and we have developed user friendly R code (https://osf.io/sz6ka/) to facilitate this. Our third main contribution is that we have studied the statistical properties of study specific prediction intervals in two simulation studies. These simulation studies have revealed a defect where the coverage probability of study specific prediction intervals is low if the between‐study variance is small but non‐negligible. These results agree with previous simulation studies for the prediction interval for the true effect in a new study.^57^, ^58^ We recommend researchers to be cautious when interpreting both types of prediction intervals when there may be a small amount of between‐study heterogeneity, as is commonly the case.

Further simulation and empirical work would be worthwhile. Simulation studies could explore a wider range of settings and simulation outcomes. For example, future simulation studies might examine the properties of study specific prediction intervals for other effect size measures than the log odds ratio that we have examined or assess the family‐wise error rate of multiple study specific prediction intervals. Another possibility is to explore the value of the proposed methodology to better accommodate any apparent outliers, by shrinking outlying results towards the pooled effect. A variety of alternative methods for handling outliers in meta‐analysis have been proposed.^60^, ^61^ However, in our simulation study, we found that the coverage probability is below the nominal level for the study with the largest true effect. Furthermore, our methodology does not alter the inferences for the average effect. We do not therefore suggest that this is a primary purpose of our methods, rather this is another possible motivation for using them. Other options for future research are examining the properties of study specific prediction intervals in arm based models and studying the best approach for testing whether two estimates of study specific true effects are different from each other.

Empirical Bayes estimates or BLUPs and corresponding study specific prediction intervals can be used in conjunction with all available estimators for the between‐study variance in true effect size. The Paule‐Mandel estimator might appear to be the most logical estimator to use, because it has been shown to be equivalent to an estimator developed in the context of empirical Bayes estimation.^47^, ^48^ However, the between‐study variance is assumed to be known in the theory of empirical Bayes estimation, so there is no restriction on computing these estimates in combination with the Paule‐Mandel estimator. Hence, researchers are advised to create empirical Bayes estimates or BLUPs and study specific prediction intervals using an estimator for the between‐study variance that is expected to perform well for their meta‐analysis.

We have focused on the univariate random‐effects model for meta‐analysis in this tutorial. It is, however, common practice to include study characteristics as moderator variables in a so‐called meta‐regression model.^30^, ^62^, ^63^ Such a meta‐regression model can be used to explain between‐study heterogeneity. The general model of Robinson^32^ can be used for obtaining the empirical Bayes estimates or BLUPs for the meta‐regression model. These estimates are then the estimates of a study specific true effect at the study's value of the moderator variable and can also be accompanied by study specific prediction intervals. By using the general model of Robinson,^32^ empirical Bayes estimates or BLUPs (and corresponding prediction intervals) can also be obtained for a multivariate meta‐analysis model where multiple dependent effect sizes are extracted from the same study.

We have presented the empirical Bayes estimates or BLUPs and corresponding study specific prediction intervals in the context of the random‐effects meta‐analysis model. However, our results readily extend to multicenter clinical trials where the effectiveness of a drug or treatment is tested in multiple medical centers. Goals of multicenter clinical trials exactly coincide with the goals of a random‐effects meta‐analysis and include estimating the effectiveness of a drug or treatment and quantifying heterogeneity in effectiveness across medical centers.^24^, ^64^, ^65^ Although shrinkage might be more controversial in multicenter clinical trials than in meta‐analysis, empirical Bayes estimates or BLUPs are also relevant in this context to obtain center specific effect size estimates that can be accompanied by a prediction interval.

The random‐effects model for meta‐analysis, and study specific prediction intervals in particular, make use of normality assumptions^16^ of which assuming known within‐study sampling variances is probably the strongest assumption. Our simulation studies revealed that violations of these assumptions adversely affected the performance of study specific prediction intervals. This calls for models that make less use of normality assumptions.^12^, ^66^ The best way to compute study specific prediction intervals when using generalized linear mixed models is an open question.

Study specific prediction intervals, and prediction intervals for the true effect in a new trial, also follow naturally from a fully Bayesian approach.^19^, ^20^ Bayesian methods also have the advantage of accommodating the uncertainty in *τ* ^2^. Further work that assesses the sensitivity of prediction intervals to the assumed prior distribution of *τ* ^2^ would be worthwhile.

To summarize, we suggest using the formula of Raudenbush and Bryk^18^ for computing study specific prediction intervals and routinely embellishing forest plots with empirical Bayes estimates and study specific prediction intervals. However, we also advise caution when interpreting the results, because we have found that prediction intervals do not perform perfectly. We hope that our tutorial serves to clarify and exemplify the methods we advocate, and also that it may promote them to the point where they might be routinely used.

### Highlights

What is already known: The pooled estimate of the average effect is of primary interest when fitting the random‐effects model for meta‐analysis. However, estimates of study specific effects are also often of interest. Empirical Bayes estimates or Best Linear Unbiased Predictions (BLUPs) of the study specific true effects are sometimes claimed to provide a better indication of a study's true effect than the more familiar study specific estimates conventionally displayed on forest plots. These estimated study specific true effects can be accompanied by prediction intervals.

What is new: We coalesce and elucidate the diffuse literature on empirical Bayes estimates and BLUPs, and show that these are the same under the random‐effects model. We reflect on the existing variance formulae for computing study specific prediction intervals. We perform simulation studies to evaluate the statistical properties of study specific prediction intervals based on different variance formulae. Our simulation studies reveal an important defect of study specific prediction intervals if between‐study variance is small, but not negligible. We present our paper as the first accessible, but statistically rigorous, account of these methods for the meta‐analysis community.

Potential impact for RSM readers outside the authors' field: The statistical methods that we advocate are slightly more sophisticated than those commonly used and our hope is that our work will make them accessible to a wider audience. Researchers are advised to be cautious when interpreting study specific prediction intervals because they do not perform perfectly. Despite this, we corroborate others by recommending the embellishment of forest plots with study specific prediction intervals.

## FINANCIAL DISCLOSURE

Robbie C.M. van Aert is supported by the European Research Council. Grant Number: 726361 (IMPROVE).

## CONFLICT OF INTEREST

The authors declare no potential conflict of interests.

## Supporting information

**AppendixS1.** Supplementary Information

## Data Availability

The data that supports the findings of this study are available in the supplementary material of this article.
