# Supplementary material for: Study specific prediction intervals for random‐effects meta‐analysis: A tutorial: Prediction intervals in meta‐analysis
Source: Res Synth Methods. 2021 Jun 3;12(4):429–47. doi: 10.1002/jrsm.1490 (PMC8361666; doi:10.1002/jrsm.1490)
Supplement: Supplementary file 1 — AppendixS1. Supplementary Information [file JRSM-12-429-s001.zip › JRSM_1490_si_sim.pdf]

Supplementary materials of 'Study specific prediction intervals for random-effects meta-analysis: A tutorial'

Robbie C.M. van Aert, Christopher H. Schmid, David Svensson, and Dan Jackson

This file contains the results of simulations for assessing the properties of the study specific prediction intervals of best linear unbiased predictions (BLUPs). The first simulation study was used for assessing properties of study specific prediction intervals using normally distributed outcome data and binary outcome data was used in the second simulation study.

Simulation study 1: Normally distributed outcome data

Results based on the Raudenbush formula (equation (10) in the paper), are presented first followed by the results based on the Quan et al. formula (equation (13) in the paper).

Results based on the Raudenbush formula

Coverage probability of the study specific prediction interval belonging to the largest θ_i in a meta-analysis

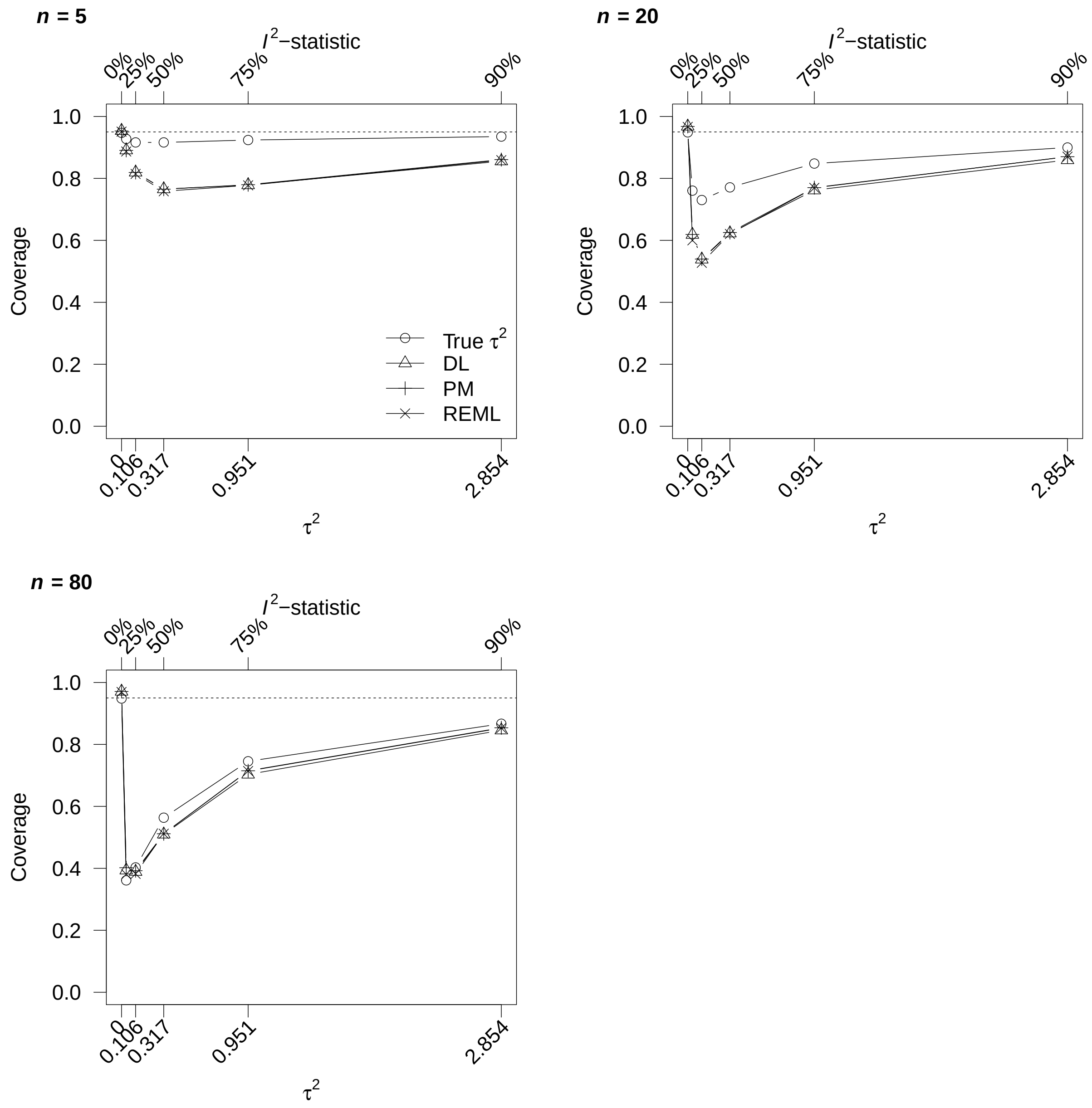

Coverage probability of the study specific prediction interval with the largest σ_i^2 in a meta-analysis

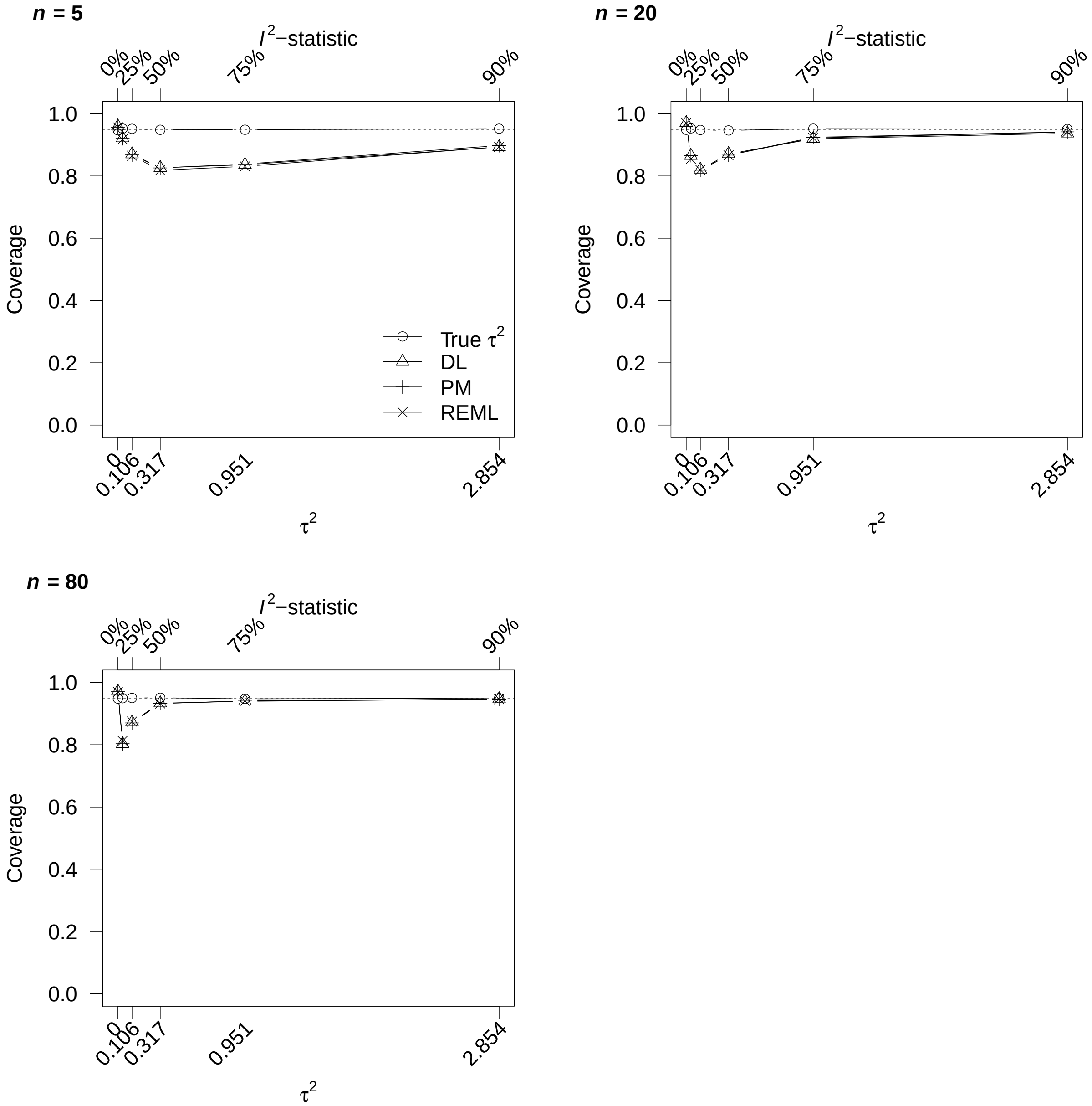

Coverage probability of study specific prediction intervals when the Knapp-Hartung adjustment was used

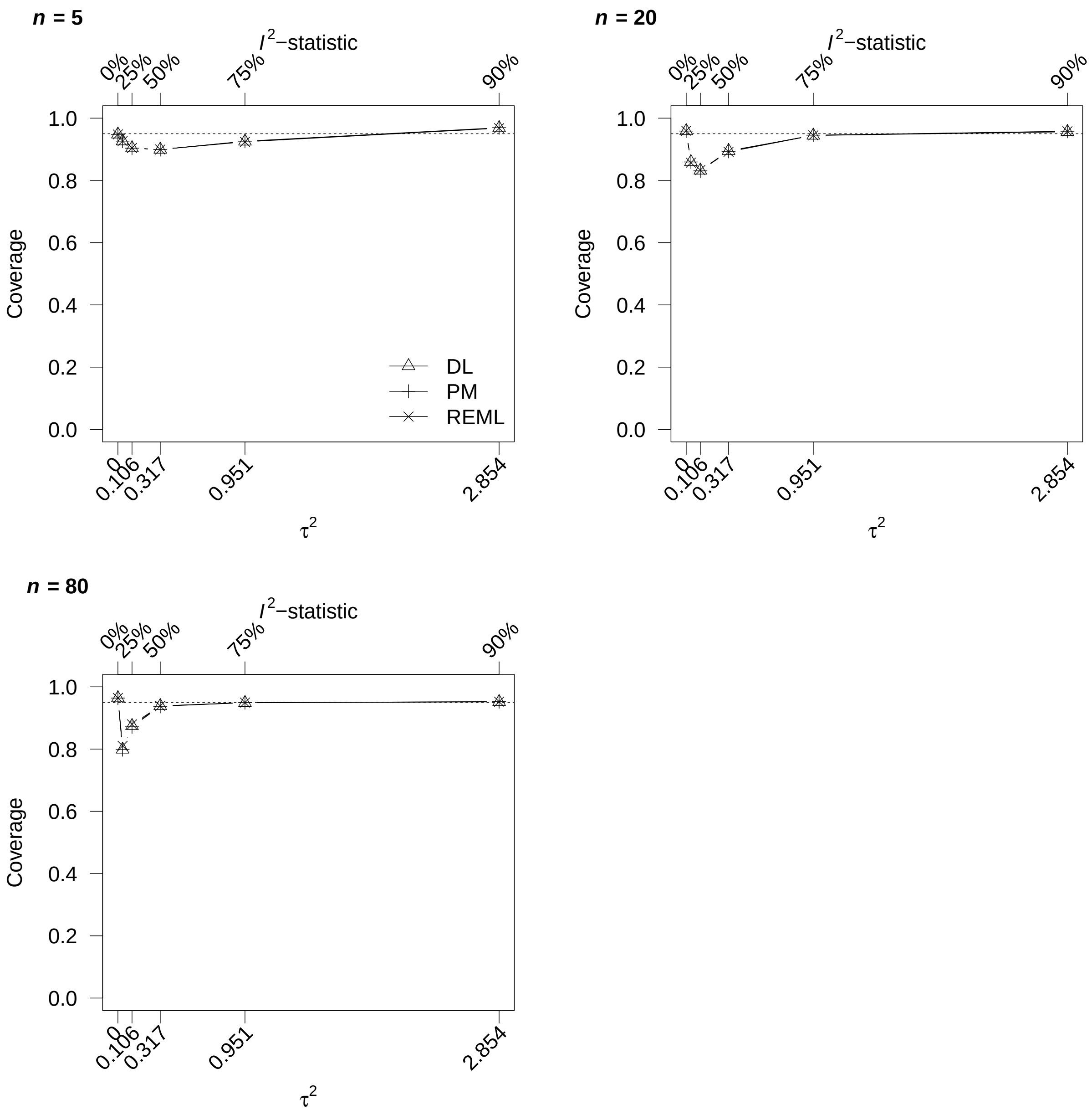

Coverage probability of the study specific prediction interval belonging to the largest θ_i in a meta-analysis when the Knapp-Hartung adjustment was used

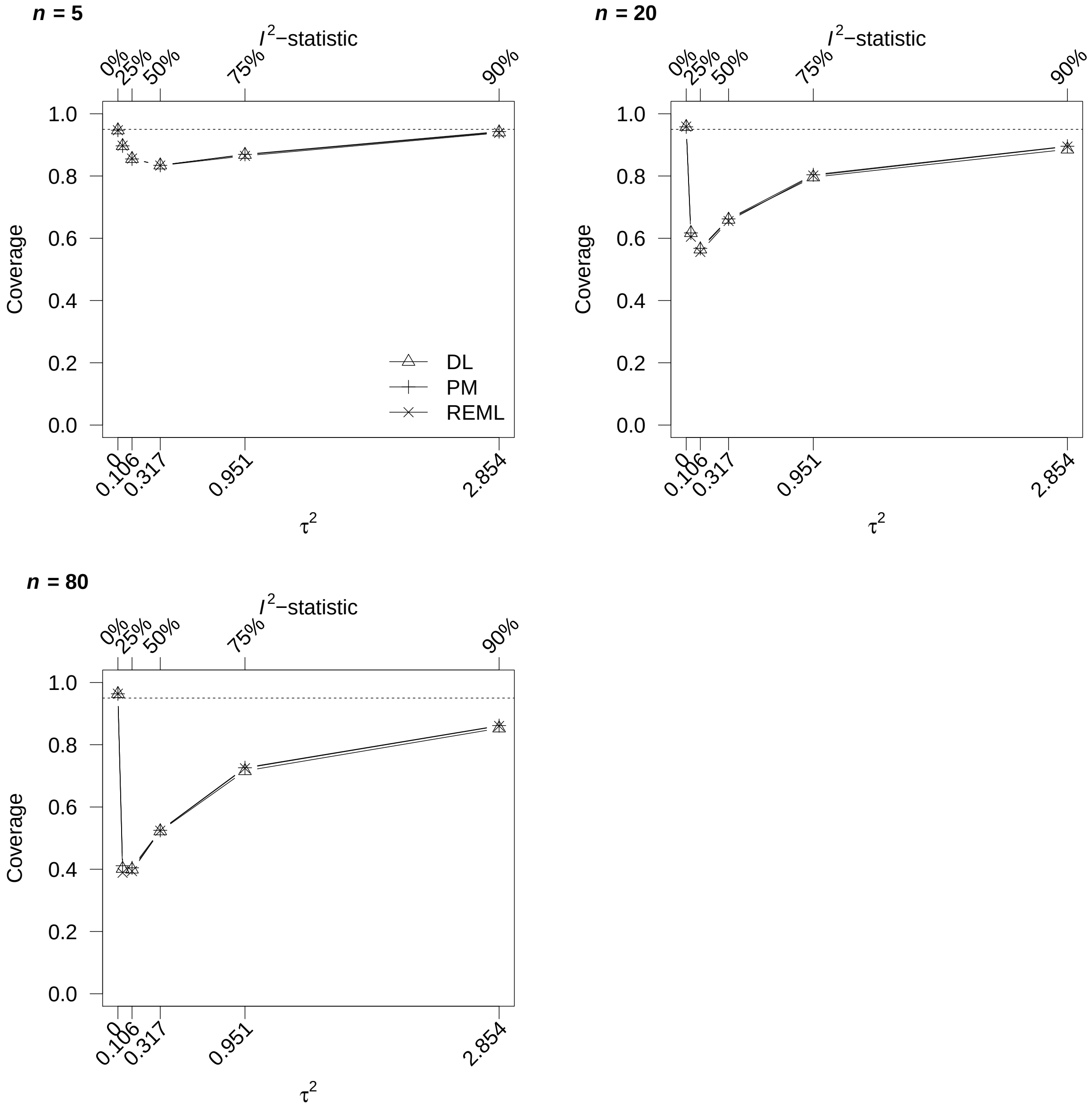

Coverage probability of the study specific prediction interval with the largest σ_i^2 in a meta-analysis when the Knapp-Hartung adjustment was used

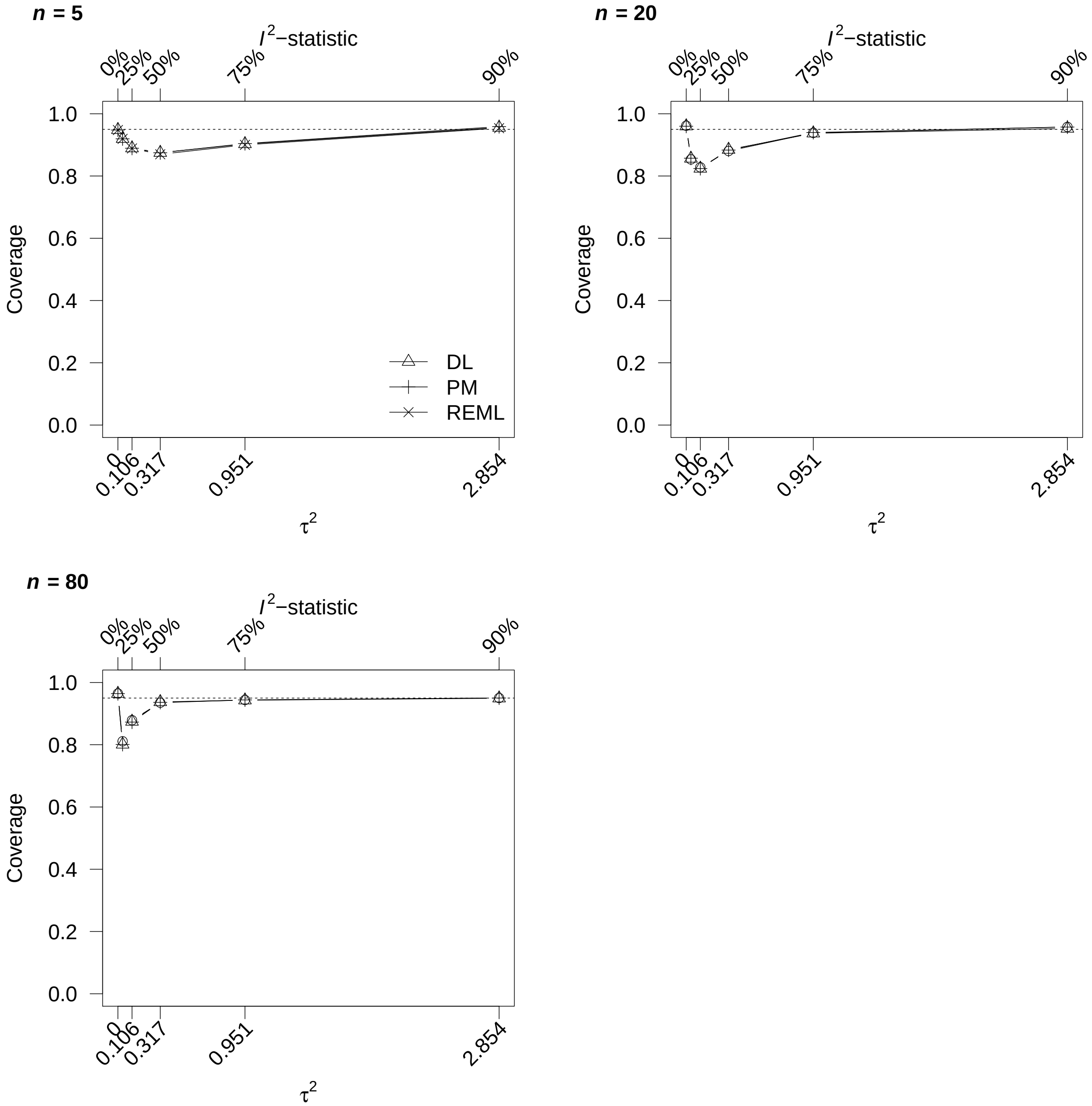

Average width of the 95% prediction intervals

- true = using known τ^2
- DL = using DerSimonian and Laird estimator
- PM = using Paule-Mandel estimator
- REML = using restricted-maximum likelihood estimator
- X_adj = when Knapp-Hartung adjustment was applied

```
##    n I2  true    DL    PM  REML DL_adj PM_adj REML_adj
##    5  0 1.057 1.313 1.327 1.300  1.606  1.622    1.593
##   20  0 0.503 0.784 0.808 0.743  0.800  0.826    0.762
##   80  0 0.248 0.487 0.503 0.447  0.487  0.503    0.447
##    5 10 1.254 1.360 1.372 1.351  1.698  1.712    1.690
##   20 10 0.860 0.889 0.911 0.862  0.923  0.946    0.899
##   80 10 0.742 0.690 0.704 0.681  0.697  0.711    0.689
##    5 25 1.504 1.445 1.456 1.437  1.855  1.868    1.847
##   20 25 1.222 1.100 1.114 1.091  1.161  1.175    1.154
##   80 25 1.148 1.065 1.071 1.066  1.081  1.087    1.083
##    5 50 1.879 1.659 1.666 1.655  2.236  2.244    2.233
##   20 50 1.709 1.575 1.579 1.568  1.680  1.684    1.673
##   80 50 1.665 1.630 1.631 1.631  1.656  1.656    1.656
##    5 75 2.265 1.995 2.000 1.992  2.780  2.785    2.776
##   20 75 2.181 2.107 2.114 2.111  2.251  2.258    2.255
##   80 75 2.156 2.137 2.141 2.141  2.170  2.174    2.174
##    5 90 2.518 2.354 2.362 2.357  3.329  3.334    3.329
##   20 90 2.487 2.449 2.458 2.458  2.617  2.625    2.625
##   80 90 2.479 2.470 2.473 2.473  2.509  2.512    2.512
```

Results based on the Quan et al. formula

Coverage probability of the study specific prediction interval

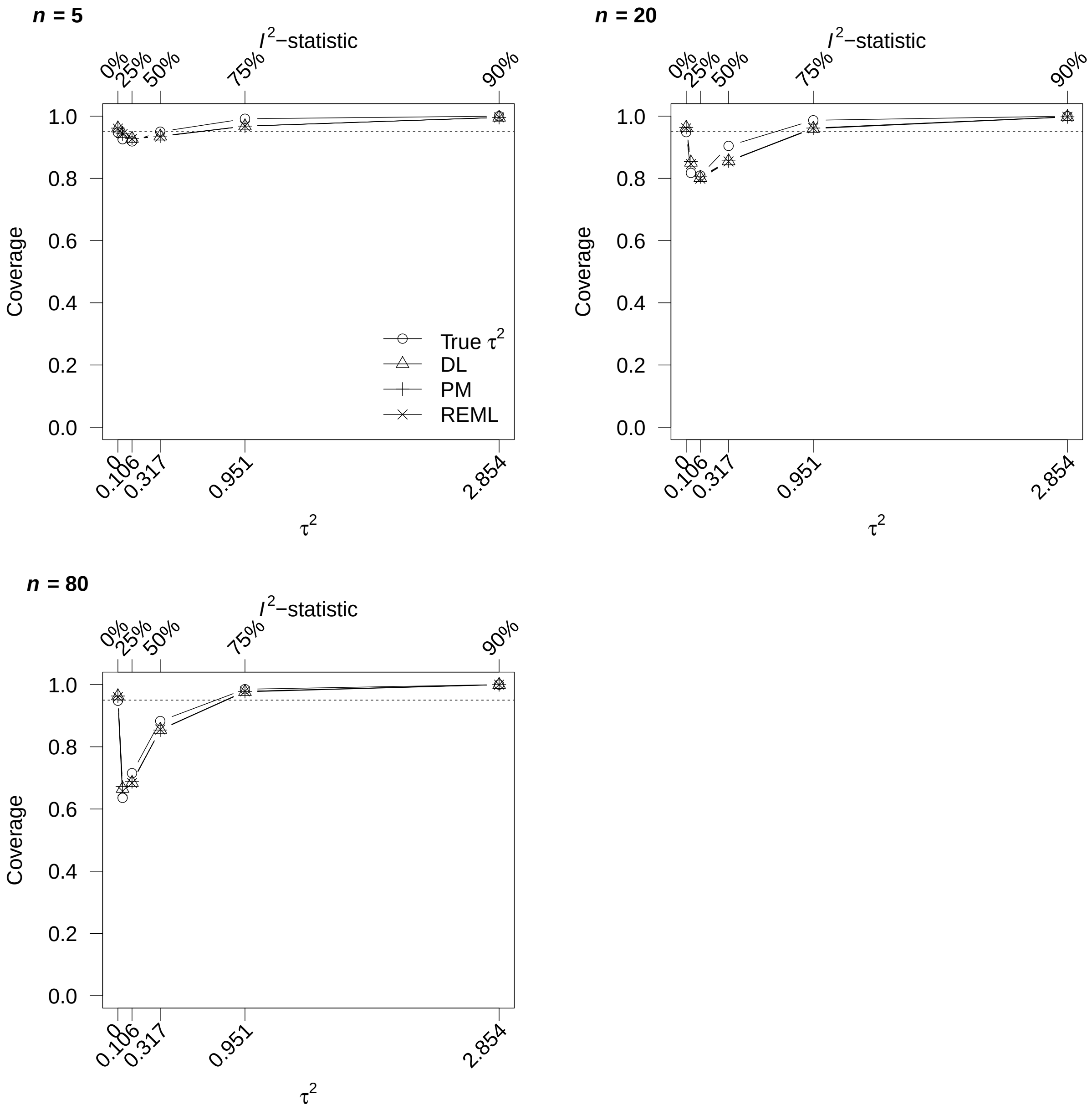

Coverage probability of the study specific prediction interval belonging to the largest θ_i in a meta-analysis

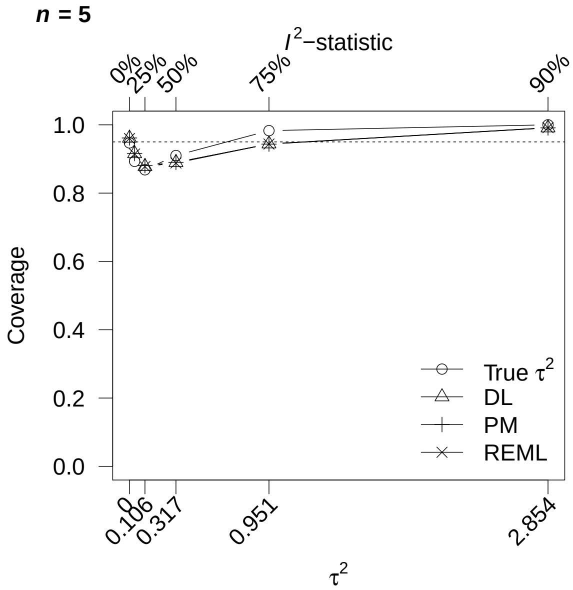

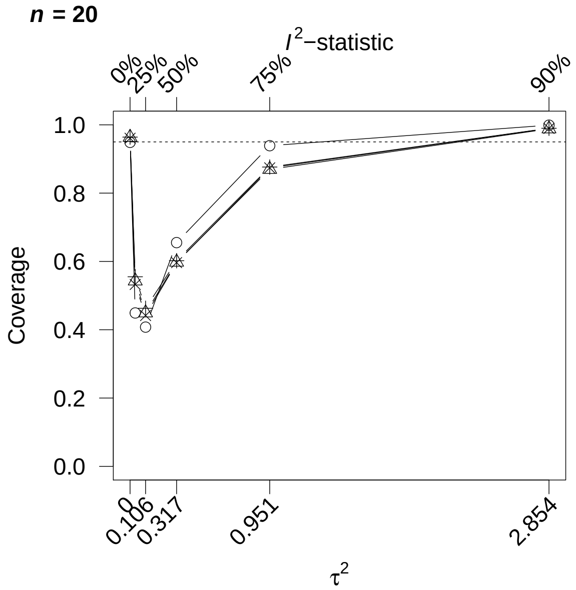

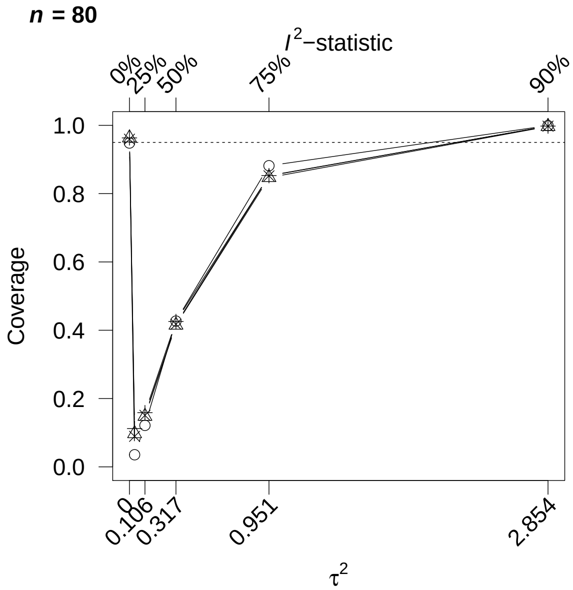

Average width of the 95% prediction intervals

- true = using known τ^2
- DL = using DerSimonian and Laird estimator
- PM = using Paule-Mandel estimator
- REML = using restricted-maximum likelihood estimator

```
##    n I2  true    DL    PM  REML
##    5  0 1.057 1.275 1.317 1.276
##   20  0 0.503 0.620 0.654 0.601
##   80  0 0.248 0.308 0.322 0.291
##    5 10 1.150 1.398 1.439 1.403
##   20 10 0.588 0.718 0.754 0.706
##   80 10 0.347 0.406 0.423 0.394
##    5 25 1.360 1.608 1.650 1.617
##   20 25 0.836 0.931 0.966 0.928
##   80 25 0.650 0.660 0.676 0.657
##    5 50 1.989 2.180 2.217 2.193
##   20 50 1.578 1.578 1.600 1.578
##   80 50 1.460 1.439 1.446 1.441
##    5 75 3.484 3.467 3.499 3.487
##   20 75 3.206 3.128 3.146 3.137
##   80 75 3.135 3.098 3.110 3.106
##    5 90 6.334 6.074 6.112 6.107
##   20 90 6.160 5.986 6.024 6.022
##   80 90 6.116 6.077 6.096 6.095
```

Simulation study 2: Binary outcome data

Results based on the Raudenbush formula, equation (10) in the paper, are presented first followed by the results based on the Quan et al. formula, equation (13) in the paper.

Results based on the Raudenbush formula

Coverage probability of the study specific prediction interval

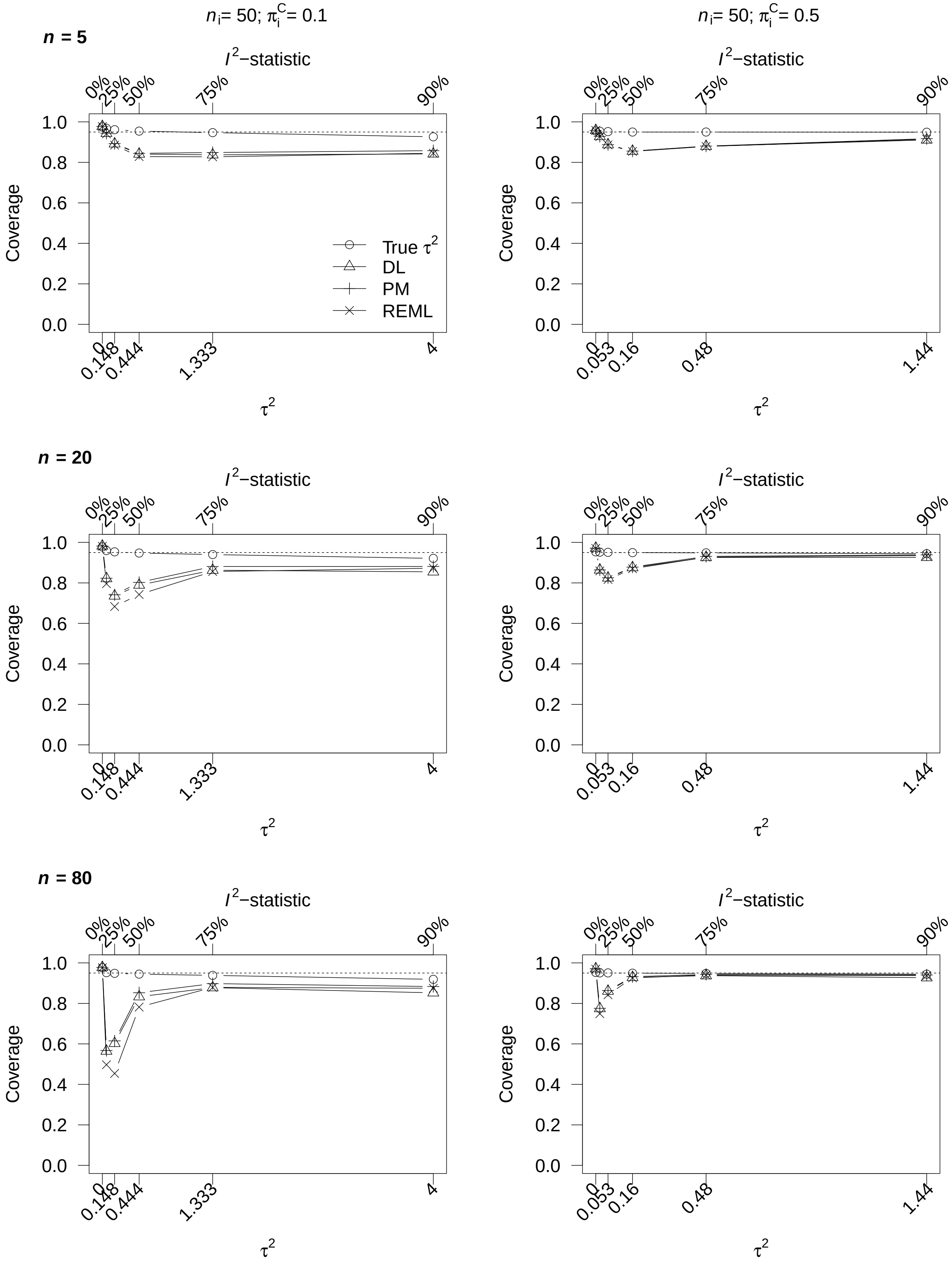

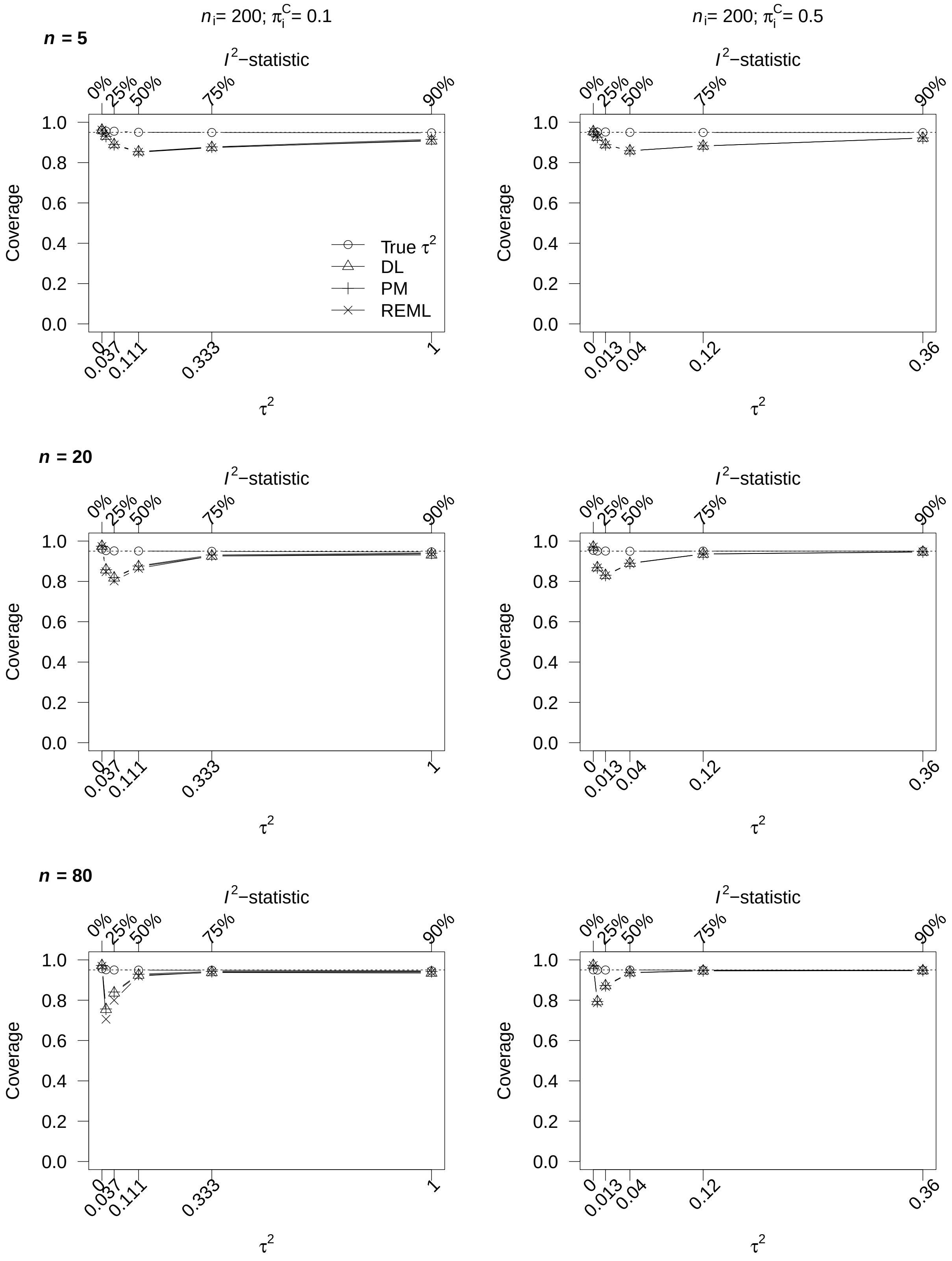

n_i= 200; π_i^C= 0.1
n_i= 200; π_i^C= 0.5
n = 5
n = 20
n = 80
I^2−statistic
0%
25%
50%
75%
90%
Coverage
1.0
0.8
0.6
0.4
0.2
0.0
True τ^2
DL
PM
REML
0
0.037
0.111
0.333
1
0.013
0.04
0.12
0.36
τ^2

Coverage probability of the study specific prediction interval belonging to the largest θ_i in a meta-analysis

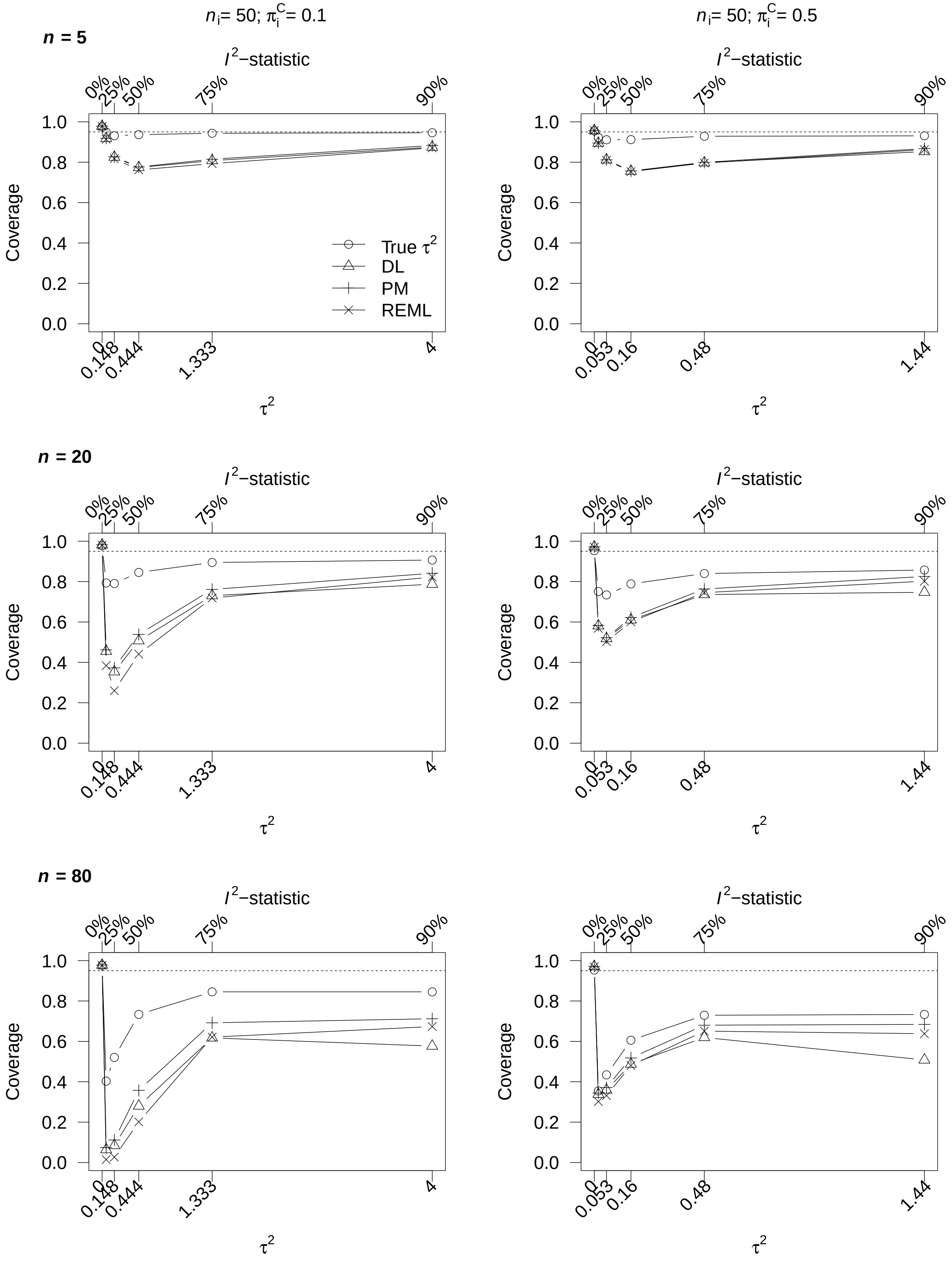

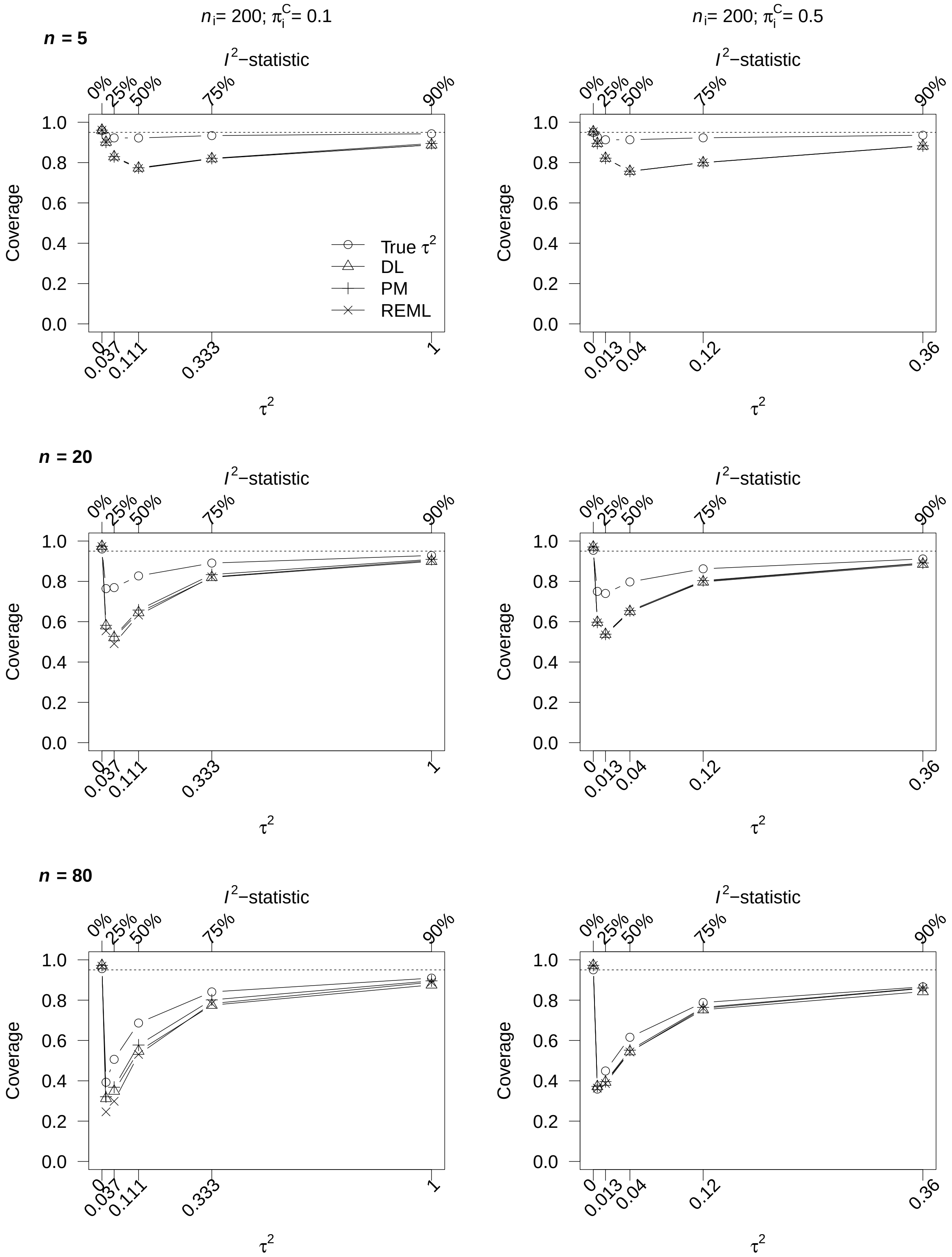
n_i= 200; π_i^C= 0.1
n_i= 200; π_i^C= 0.5
n = 5
n = 20
n = 80
I^2−statistic
0% 25% 50% 75% 90%
Coverage
1.0 0.8 0.6 0.4 0.2 0.0
True τ^2
DL
PM
REML
0 0.037 0.111 0.333 1
0 0.013 0.04 0.12 0.36
τ^2

Coverage probability of the study specific prediction interval when the Knapp-Hartung adjustment was used

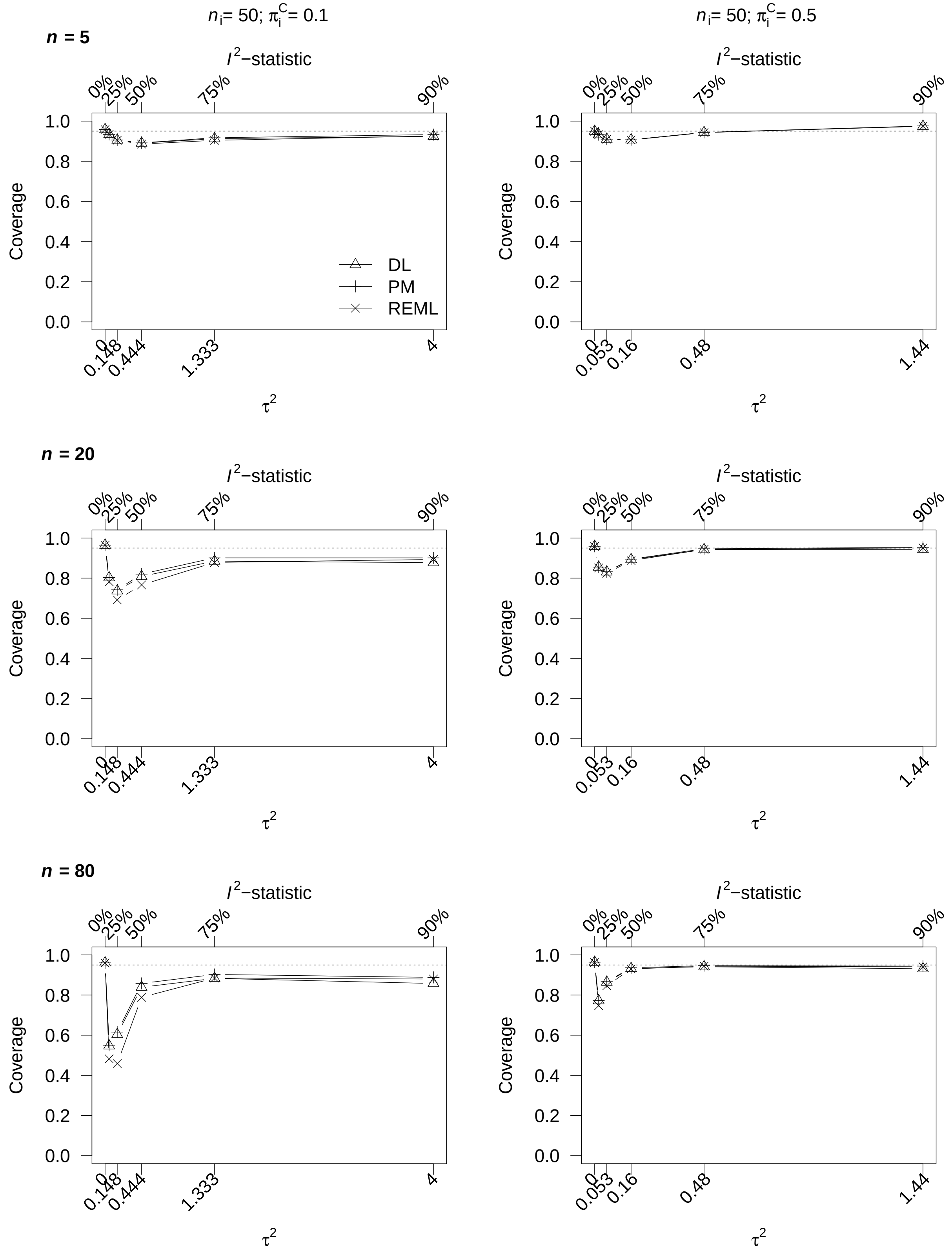

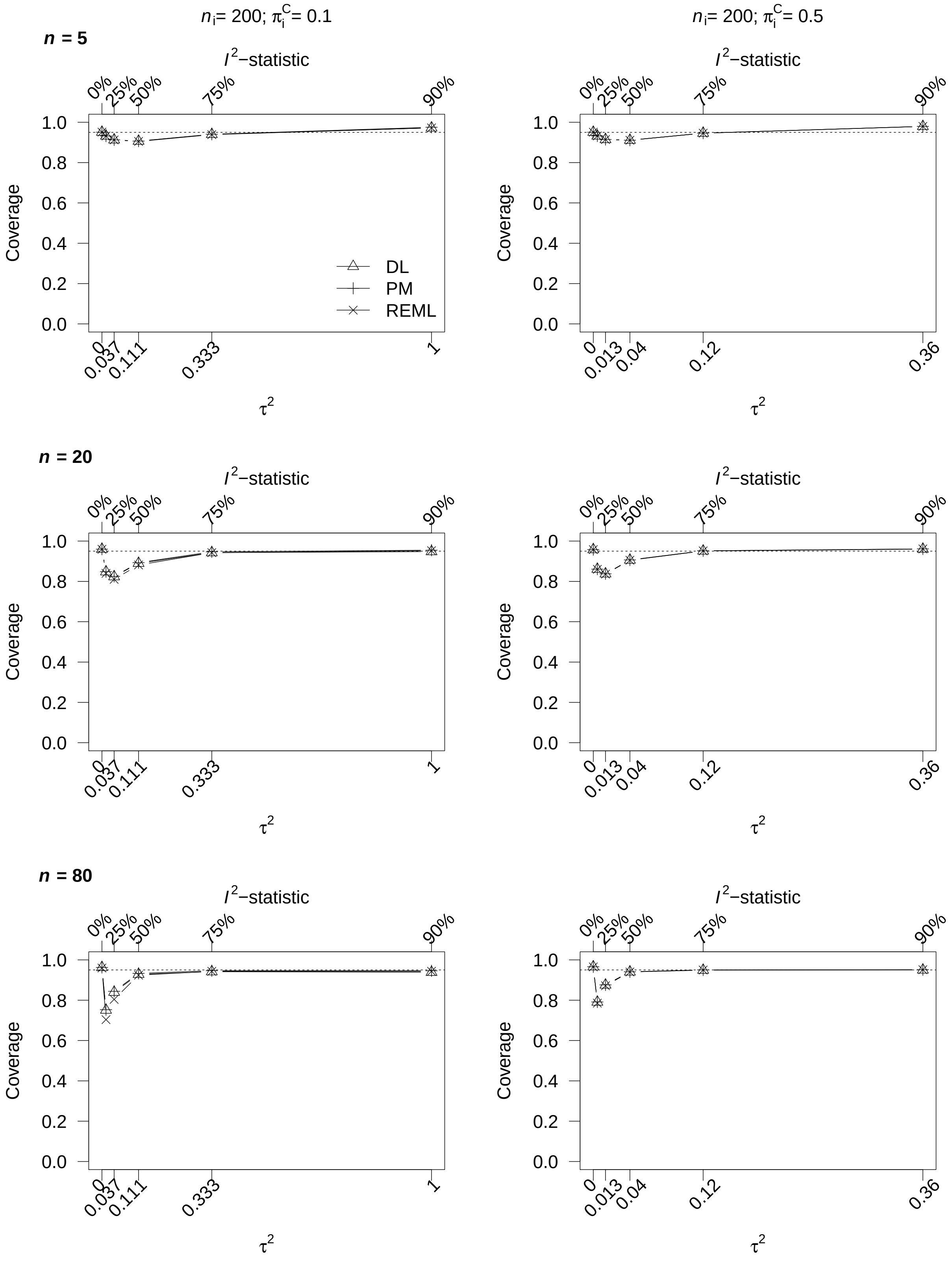

n_i= 200; π_i^C= 0.1
n_i= 200; π_i^C= 0.5
n = 5
n = 20
n = 80
I^2−statistic
Coverage
τ^2
DL
PM
REML

Coverage probability of the study specific prediction interval belonging to the largest θ_i in a meta-analysis when the Knapp-Hartung adjustment was used

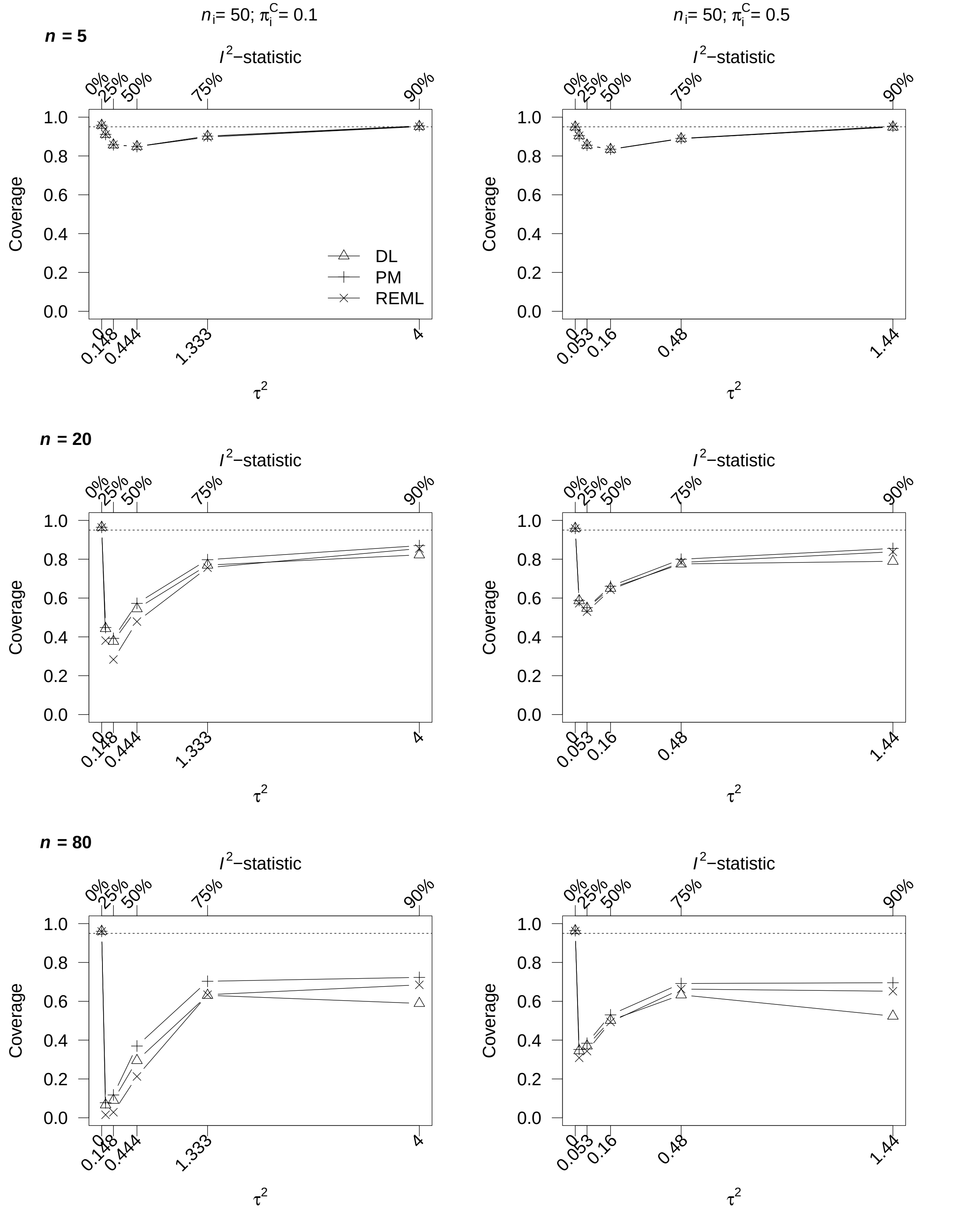

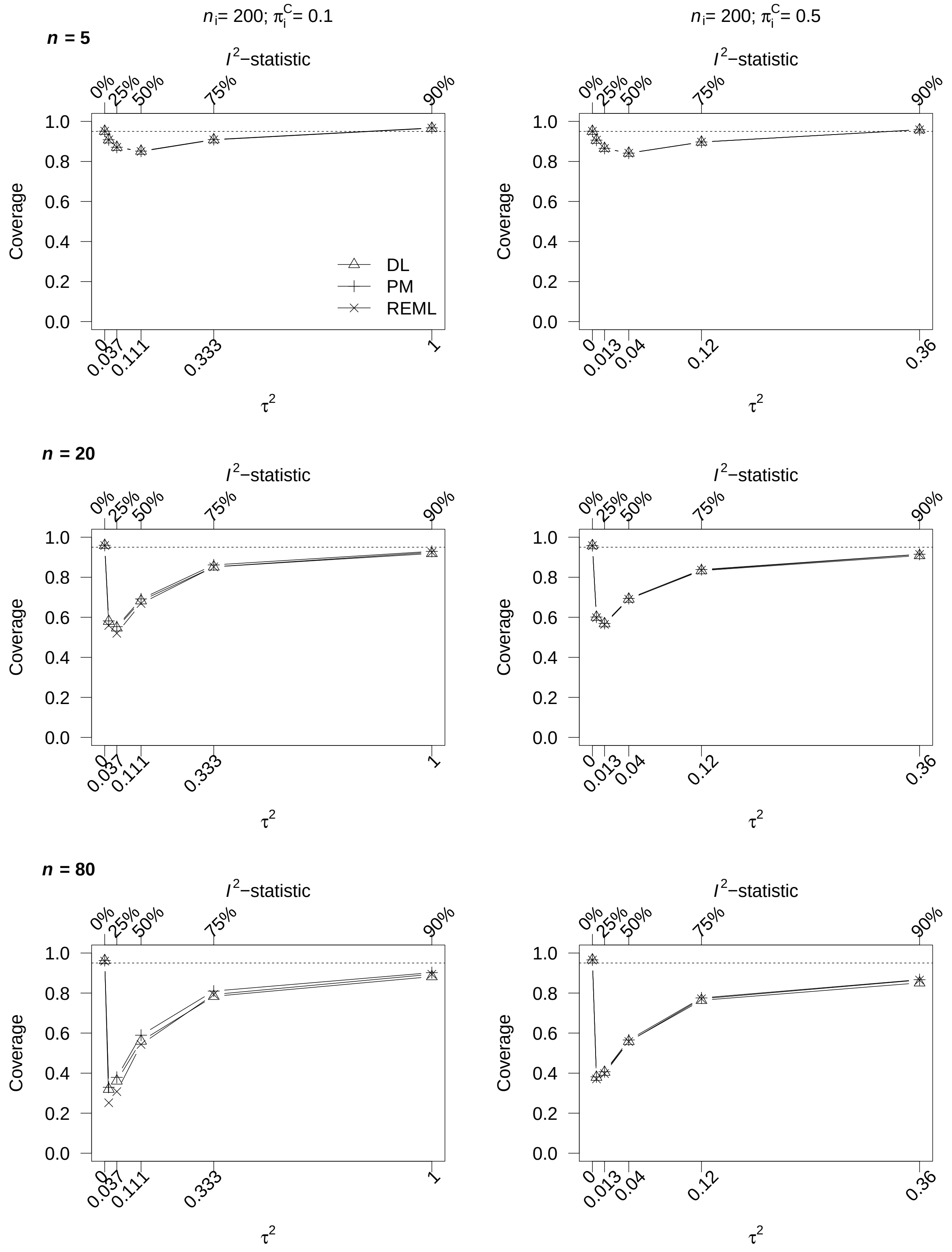

n_i= 200; π_i^C= 0.1
n_i= 200; π_i^C= 0.5
n = 5
n = 20
n = 80
I^2−statistic
0% 25% 50% 75% 90%
Coverage
1.0 0.8 0.6 0.4 0.2 0.0
DL
PM
REML
0 0.037 0.111 0.333 1
0 0.013 0.04 0.12 0.36
τ^2

Average width of the 95% prediction intervals

- true = using known τ^2
- DL = using DerSimonian and Laird estimator
- PM = using Paule-Mandel estimator
- REML = using restricted-maximum likelihood estimator
- X_adj = when Knapp-Hartung adjustment was applied

```
##   n   I2 pics  ni  true    DL    PM  REML DL_adj PM_adj REML_adj
##   5 0.00  0.1  50 1.171 1.350 1.364 1.321  1.609  1.625    1.580
##  20 0.00  0.1  50 0.583 0.698 0.709 0.643  0.678  0.689    0.623
##  80 0.00  0.1  50 0.291 0.322 0.324 0.295  0.300  0.303    0.274
##   5 0.10  0.1  50 1.391 1.393 1.410 1.361  1.703  1.722    1.670
##  20 0.10  0.1  50 0.996 0.776 0.792 0.700  0.779  0.795    0.703
##  80 0.10  0.1  50 0.872 0.399 0.408 0.318  0.389  0.398    0.309
##   5 0.25  0.1  50 1.667 1.473 1.494 1.441  1.869  1.893    1.836
##  20 0.25  0.1  50 1.407 0.964 0.992 0.857  1.003  1.032    0.897
##  80 0.25  0.1  50 1.334 0.706 0.734 0.500  0.713  0.741    0.507
##   5 0.50  0.1  50 2.073 1.708 1.740 1.671  2.303  2.339    2.266
##  20 0.50  0.1  50 1.925 1.479 1.529 1.382  1.577  1.628    1.481
##  80 0.50  0.1  50 1.887 1.509 1.570 1.397  1.533  1.594    1.422
##   5 0.75  0.1  50 2.505 2.103 2.148 2.078  2.943  2.992    2.918
##  20 0.75  0.1  50 2.424 2.129 2.195 2.118  2.277  2.344    2.266
##  80 0.75  0.1  50 2.404 2.152 2.224 2.159  2.186  2.259    2.194
##   5 0.90  0.1  50 2.961 2.611 2.663 2.621  3.698  3.752    3.707
##  20 0.90  0.1  50 2.912 2.655 2.744 2.715  2.841  2.930    2.901
##  80 0.90  0.1  50 2.901 2.649 2.751 2.722  2.692  2.794    2.765
##   5 0.00  0.5  50 0.701 0.842 0.843 0.840  1.020  1.021    1.019
##  20 0.00  0.5  50 0.351 0.517 0.519 0.507  0.524  0.526    0.515
##  80 0.00  0.5  50 0.175 0.315 0.317 0.295  0.313  0.315    0.293
##   5 0.10  0.5  50 0.830 0.875 0.876 0.873  1.091  1.092    1.090
##  20 0.10  0.5  50 0.597 0.586 0.589 0.575  0.606  0.609    0.596
##  80 0.10  0.5  50 0.523 0.446 0.450 0.420  0.450  0.454    0.424
##   5 0.25  0.5  50 0.994 0.929 0.931 0.928  1.198  1.200    1.197
##  20 0.25  0.5  50 0.842 0.732 0.737 0.721  0.772  0.777    0.761
##  80 0.25  0.5  50 0.799 0.714 0.721 0.690  0.724  0.731    0.701
##   5 0.50  0.5  50 1.223 1.066 1.069 1.065  1.444  1.447    1.443
##  20 0.50  0.5  50 1.142 1.030 1.040 1.024  1.099  1.109    1.093
##  80 0.50  0.5  50 1.121 1.072 1.086 1.068  1.089  1.103    1.085
##   5 0.75  0.5  50 1.439 1.297 1.304 1.300  1.818  1.826    1.821
##  20 0.75  0.5  50 1.402 1.342 1.361 1.351  1.434  1.454    1.443
##  80 0.75  0.5  50 1.393 1.353 1.376 1.365  1.374  1.397    1.386
##   5 0.90  0.5  50 1.651 1.565 1.585 1.579  2.222  2.241    2.234
##  20 0.90  0.5  50 1.634 1.582 1.616 1.606  1.691  1.726    1.716
##  80 0.90  0.5  50 1.630 1.580 1.618 1.608  1.605  1.643    1.633
##   5 0.00  0.1 200 0.586 0.698 0.700 0.696  0.846  0.848    0.843
##  20 0.00  0.1 200 0.292 0.414 0.418 0.399  0.418  0.422    0.403
##  80 0.00  0.1 200 0.146 0.242 0.244 0.213  0.239  0.242    0.211
##   5 0.10  0.1 200 0.693 0.725 0.728 0.723  0.903  0.905    0.900
##  20 0.10  0.1 200 0.498 0.474 0.479 0.458  0.490  0.494    0.473
##  80 0.10  0.1 200 0.436 0.350 0.355 0.309  0.352  0.358    0.312
##   5 0.25  0.1 200 0.829 0.773 0.776 0.770  0.997  1.000    0.994
##  20 0.25  0.1 200 0.701 0.597 0.604 0.580  0.630  0.637    0.612
##  80 0.25  0.1 200 0.666 0.564 0.574 0.528  0.573  0.582    0.536
##   5 0.50  0.1 200 1.019 0.887 0.891 0.885  1.203  1.208    1.201
```

```
##   20 0.50   0.1 200 0.951 0.850 0.861 0.841  0.908  0.919    0.898
##   80 0.50   0.1 200 0.934 0.882 0.896 0.875  0.896  0.910    0.889
##    5 0.75   0.1 200 1.196 1.071 1.079 1.072  1.501  1.509    1.502
##   20 0.75   0.1 200 1.164 1.114 1.128 1.117  1.190  1.205    1.194
##   80 0.75   0.1 200 1.157 1.124 1.140 1.130  1.142  1.158    1.148
##    5 0.90   0.1 200 1.350 1.273 1.290 1.280  1.807  1.824    1.814
##   20 0.90   0.1 200 1.335 1.300 1.320 1.312  1.390  1.409    1.402
##   80 0.90   0.1 200 1.331 1.302 1.321 1.315  1.323  1.341    1.335
##    5 0.00   0.5 200 0.351 0.423 0.423 0.423  0.516  0.516    0.516
##   20 0.00   0.5 200 0.175 0.262 0.262 0.261  0.266  0.266    0.265
##   80 0.00   0.5 200 0.088 0.166 0.167 0.164  0.166  0.166    0.163
##    5 0.10   0.5 200 0.415 0.435 0.436 0.435  0.543  0.543    0.543
##   20 0.10   0.5 200 0.299 0.301 0.301 0.299  0.312  0.312    0.311
##   80 0.10   0.5 200 0.262 0.235 0.235 0.232  0.237  0.238    0.234
##    5 0.25   0.5 200 0.496 0.465 0.465 0.465  0.601  0.601    0.601
##   20 0.25   0.5 200 0.420 0.374 0.374 0.373  0.395  0.395    0.393
##   80 0.25   0.5 200 0.399 0.366 0.367 0.364  0.372  0.373    0.369
##    5 0.50   0.5 200 0.608 0.535 0.536 0.535  0.727  0.728    0.727
##   20 0.50   0.5 200 0.569 0.527 0.528 0.527  0.562  0.564    0.562
##   80 0.50   0.5 200 0.559 0.545 0.547 0.545  0.554  0.555    0.554
##    5 0.75   0.5 200 0.706 0.641 0.642 0.642  0.899  0.900    0.899
##   20 0.75   0.5 200 0.689 0.671 0.673 0.672  0.716  0.719    0.718
##   80 0.75   0.5 200 0.684 0.678 0.680 0.679  0.688  0.691    0.690
##    5 0.90   0.5 200 0.768 0.735 0.737 0.736  1.040  1.041    1.041
##   20 0.90   0.5 200 0.762 0.753 0.756 0.756  0.805  0.808    0.807
##   80 0.90   0.5 200 0.760 0.755 0.759 0.758  0.767  0.770    0.770
```

Results based on the Quan et al. formula

Coverage probability of the study specific prediction interval

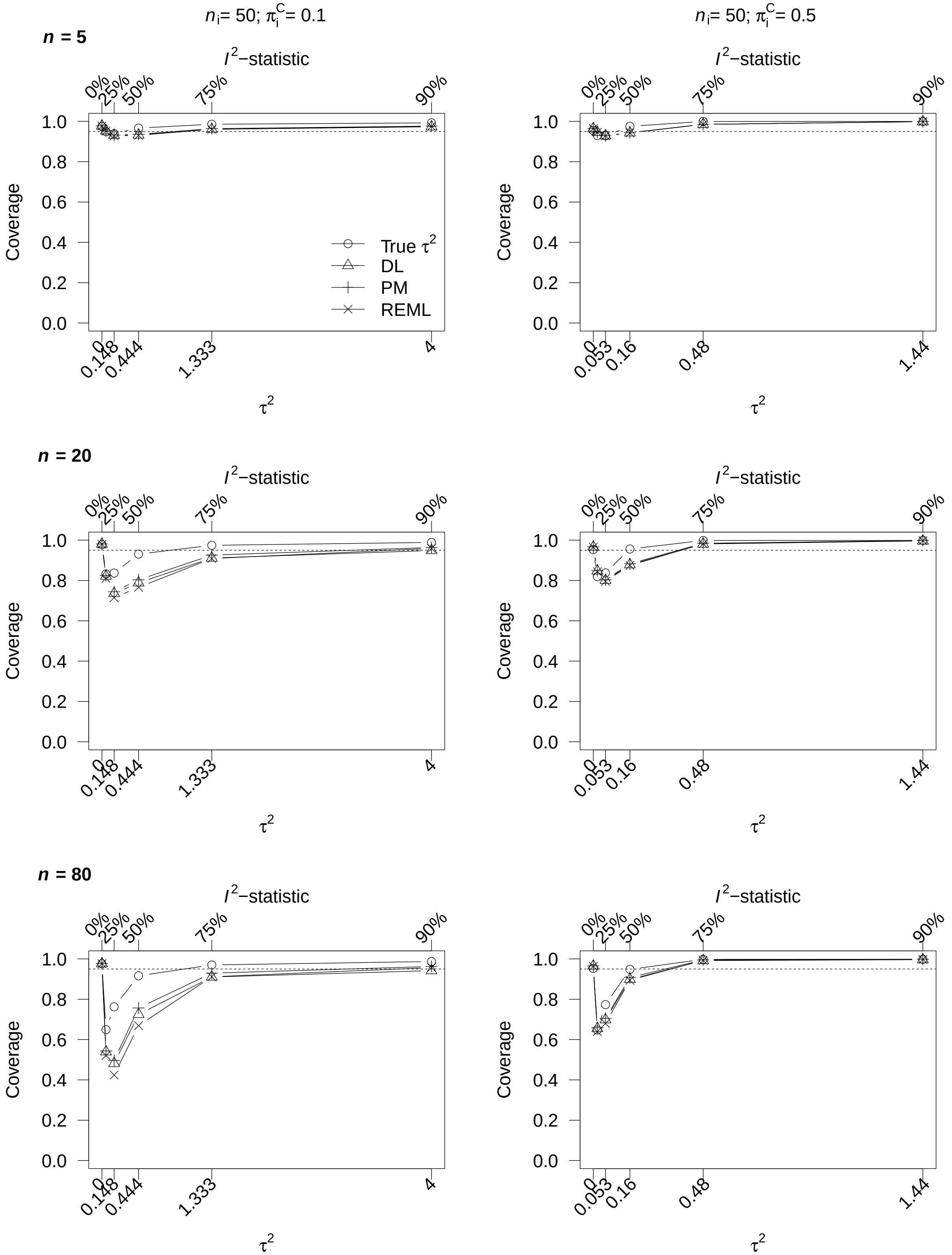

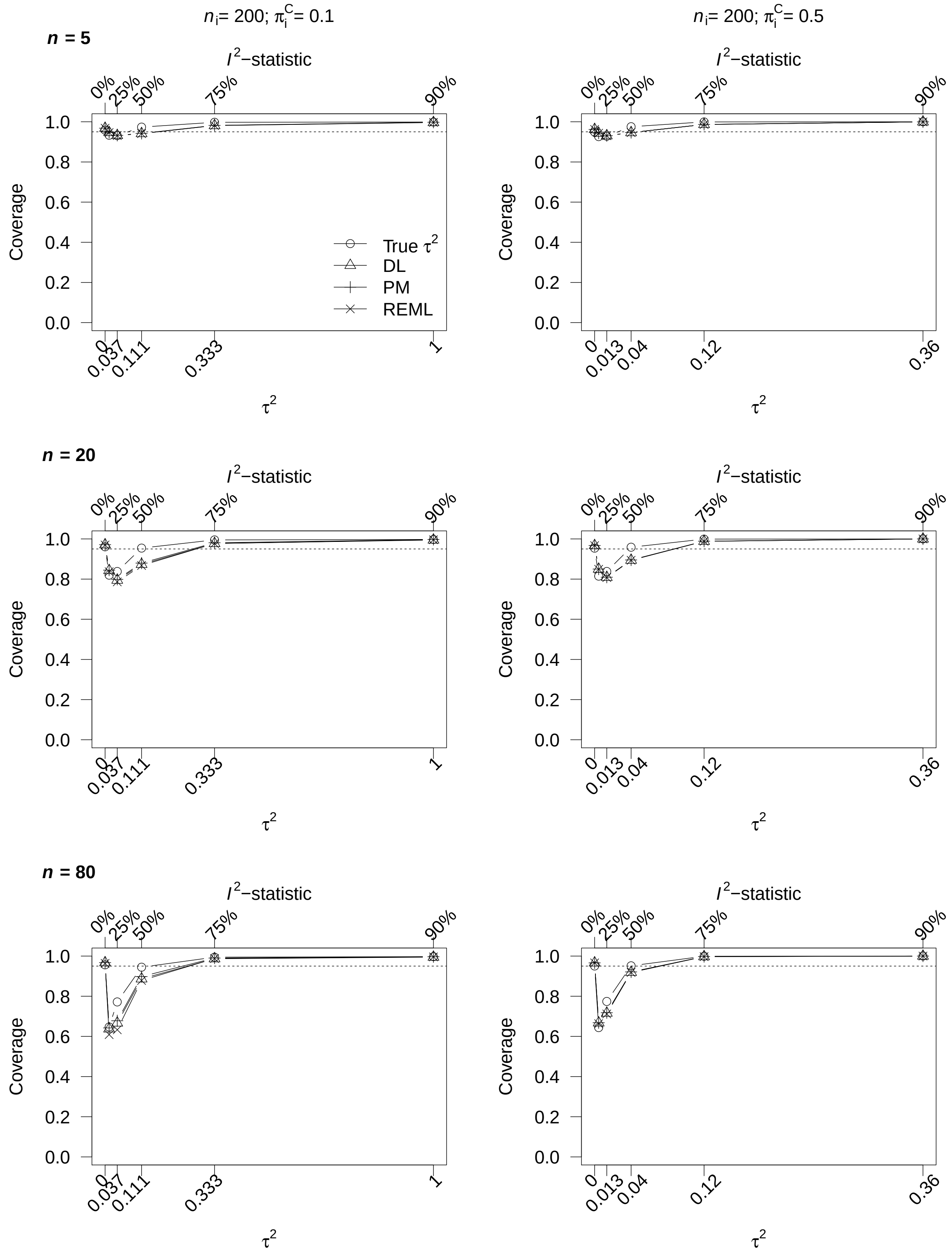

n_i= 200; π_i^C= 0.1
n_i= 200; π_i^C= 0.5
n = 5
n = 20
n = 80
I^2−statistic
0% 25% 50% 75% 90%
Coverage
0.0 0.2 0.4 0.6 0.8 1.0
True τ^2
DL
PM
REML
0 0.037 0.111 0.333 1
0 0.013 0.04 0.12 0.36
τ^2

Coverage probability of the study specific prediction interval belonging to the largest θ_i in a meta-analysis

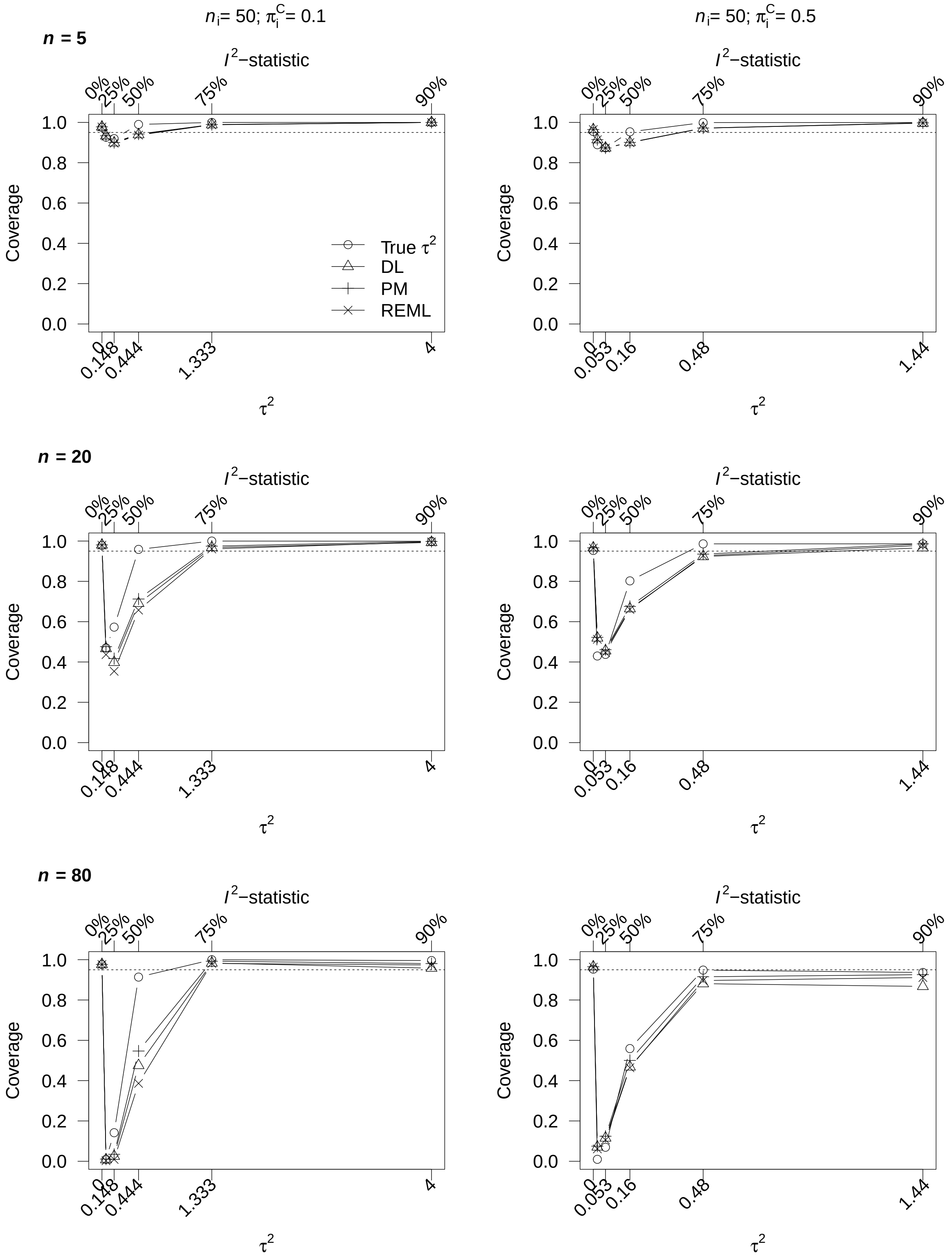

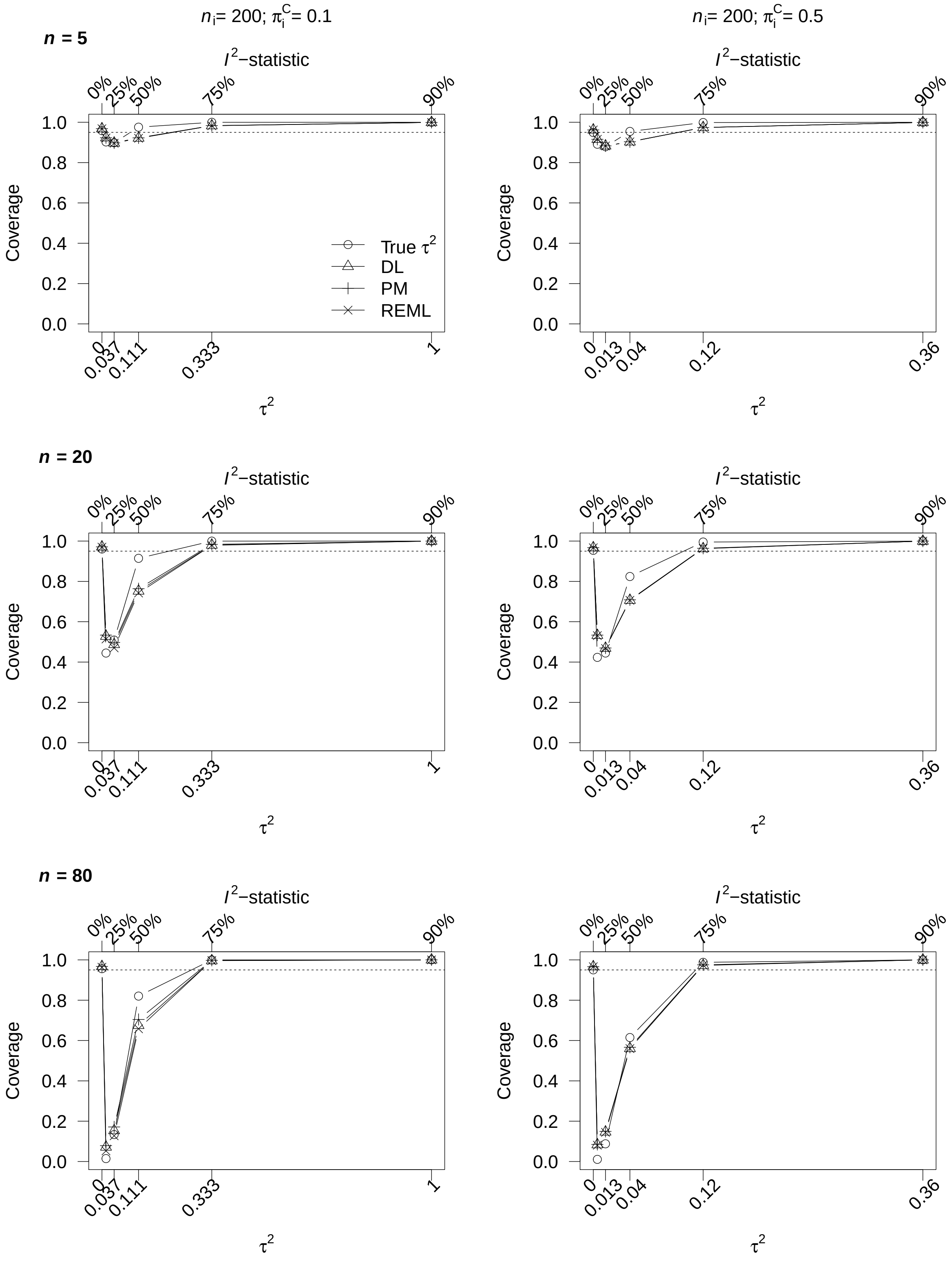

n_i= 200; π_i^C= 0.1
n_i= 200; π_i^C= 0.5
n = 5
n = 20
n = 80
I^2−statistic
0% 25% 50% 75% 90%
Coverage
1.0 0.8 0.6 0.4 0.2 0.0
True τ^2
DL
PM
REML
0 0.037 0.111 0.333 1
0 0.013 0.04 0.12 0.36
τ^2

Average width of the 95% prediction intervals

- true = using known τ^2
- DL = using DerSimonian and Laird estimator
- PM = using Paule-Mandel estimator
- REML = using restricted-maximum likelihood estimator

```
##    n   I2 pics  ni  true    DL    PM  REML
##    5 0.00  0.1  50 1.171 1.299 1.329 1.283
##   20 0.00  0.1  50 0.583 0.619 0.628 0.601
##   80 0.00  0.1  50 0.291 0.296 0.297 0.292
##    5 0.10  0.1  50 1.264 1.402 1.440 1.385
##   20 0.10  0.1  50 0.673 0.683 0.699 0.655
##   80 0.10  0.1  50 0.411 0.329 0.333 0.313
##    5 0.25  0.1  50 1.520 1.597 1.649 1.583
##   20 0.25  0.1  50 0.995 0.836 0.868 0.788
##   80 0.25  0.1  50 0.813 0.454 0.475 0.387
##    5 0.50  0.1  50 2.337 2.169 2.267 2.163
##   20 0.50  0.1  50 1.947 1.386 1.481 1.321
##   80 0.50  0.1  50 1.837 1.131 1.237 1.008
##    5 0.75  0.1  50 4.161 3.394 3.580 3.452
##   20 0.75  0.1  50 3.885 2.740 2.999 2.774
##   80 0.75  0.1  50 3.814 2.626 2.920 2.666
##    5 0.90  0.1  50 7.488 5.566 5.989 5.876
##   20 0.90  0.1  50 7.279 4.802 5.543 5.316
##   80 0.90  0.1  50 7.226 4.645 5.489 5.229
##    5 0.00  0.5  50 0.701 0.833 0.836 0.833
##   20 0.00  0.5  50 0.351 0.427 0.429 0.422
##   80 0.00  0.5  50 0.175 0.213 0.214 0.207
##    5 0.10  0.5  50 0.755 0.913 0.917 0.913
##   20 0.10  0.5  50 0.403 0.490 0.495 0.485
##   80 0.10  0.5  50 0.247 0.276 0.279 0.265
##    5 0.25  0.5  50 0.907 1.054 1.061 1.056
##   20 0.25  0.5  50 0.599 0.637 0.646 0.630
##   80 0.25  0.5  50 0.492 0.466 0.475 0.448
##    5 0.50  0.5  50 1.402 1.448 1.467 1.457
##   20 0.50  0.5  50 1.185 1.093 1.125 1.093
##   80 0.50  0.5  50 1.124 1.027 1.065 1.019
##    5 0.75  0.5  50 2.520 2.346 2.434 2.410
##   20 0.75  0.5  50 2.380 2.100 2.245 2.178
##   80 0.75  0.5  50 2.344 2.079 2.244 2.163
##    5 0.90  0.5  50 4.535 3.824 4.226 4.149
##   20 0.90  0.5  50 4.437 3.557 4.153 3.984
##   80 0.90  0.5  50 4.412 3.515 4.163 3.974
##    5 0.00  0.1 200 0.586 0.689 0.696 0.689
##   20 0.00  0.1 200 0.292 0.345 0.349 0.337
##   80 0.00  0.1 200 0.146 0.170 0.172 0.162
##    5 0.10  0.1 200 0.630 0.754 0.761 0.753
##   20 0.10  0.1 200 0.336 0.396 0.402 0.387
##   80 0.10  0.1 200 0.206 0.218 0.222 0.202
##    5 0.25  0.1 200 0.756 0.874 0.885 0.875
##   20 0.25  0.1 200 0.499 0.517 0.530 0.506
##   80 0.25  0.1 200 0.410 0.359 0.371 0.334
##    5 0.50  0.1 200 1.169 1.194 1.218 1.203
##   20 0.50  0.1 200 0.986 0.890 0.923 0.885
```

```
## 80 0.50  0.1 200 0.935 0.827 0.864 0.814
##  5 0.75  0.1 200 2.099 1.934 2.008 1.979
## 20 0.75  0.1 200 1.982 1.753 1.853 1.792
## 80 0.75  0.1 200 1.951 1.742 1.850 1.783
##  5 0.90  0.1 200 3.779 3.256 3.502 3.433
## 20 0.90  0.1 200 3.698 3.153 3.473 3.360
## 80 0.90  0.1 200 3.678 3.146 3.474 3.359
##  5 0.00  0.5 200 0.351 0.419 0.420 0.419
## 20 0.00  0.5 200 0.175 0.217 0.217 0.216
## 80 0.00  0.5 200 0.088 0.110 0.110 0.109
##  5 0.10  0.5 200 0.377 0.454 0.455 0.454
## 20 0.10  0.5 200 0.202 0.251 0.251 0.250
## 80 0.10  0.5 200 0.124 0.144 0.144 0.143
##  5 0.25  0.5 200 0.453 0.529 0.530 0.529
## 20 0.25  0.5 200 0.299 0.328 0.329 0.327
## 80 0.25  0.5 200 0.247 0.243 0.245 0.241
##  5 0.50  0.5 200 0.701 0.741 0.743 0.742
## 20 0.50  0.5 200 0.594 0.577 0.581 0.578
## 80 0.50  0.5 200 0.564 0.545 0.550 0.545
##  5 0.75  0.5 200 1.263 1.226 1.236 1.234
## 20 0.75  0.5 200 1.197 1.140 1.159 1.152
## 80 0.75  0.5 200 1.179 1.142 1.164 1.156
##  5 0.90  0.5 200 2.280 2.117 2.168 2.164
## 20 0.90  0.5 200 2.239 2.091 2.183 2.171
## 80 0.90  0.5 200 2.229 2.104 2.208 2.194
```
