# Supplementary material for: Study specific prediction intervals for random‐effects meta‐analysis: A tutorial: Prediction intervals in meta‐analysis
Source: Res Synth Methods. 2021 Jun 3;12(4):429–47. doi: 10.1002/jrsm.1490 (PMC8361666; doi:10.1002/jrsm.1490)
Supplement: Supplementary file 1 — AppendixS1. Supplementary Information [file JRSM-12-429-s001.zip › JRSM_1490_supplements_analytical.pdf]

Received: Added at production | Revised: Added at production | Accepted: Added at production
DOI: xxx/xxxx

ARTICLE TYPE

Analytical Appendix of "Study specific prediction intervals for random-effects meta-analysis: A tutorial"

Summary

This Appendix proves that Robinson's equation provides the BLUPs and derives equations 13-15 of the paper. Please see the paper for more details.

DERIVATION OF BLUP FOR γ_i USING ROBINSON'S EQUATION

We show that Robinson's equation simplifies to the BLUP provided in the paper, under the random-effects for meta-analysis. As explained in the paper, the random-effects model for meta-analysis is obtained by taking $\mathbf{X} = \mathbf{1}$ (the column vector of length n where every entry is one), $\mathbf{Z} = \mathbf{G} = \mathbf{I}$ (the $n \times n$ identity matrix), $\boldsymbol{\beta} = \theta$, $\sigma^2 = \tau^2$, $\mathbf{u}$ is the column vector of length n containing the γ_i, and $\mathbf{R}$ =diag(σ_i^2/τ^2) (the $n \times n$ diagonal matrix containing the ratios of the σ_i^2 and τ^2). In his more general model, Robinson gives the estimating equation (as his equation 1.2)

$$\mathbf{Z}^T\mathbf{R}^{-1}\mathbf{X}\hat{\boldsymbol{\beta}} + (\mathbf{Z}^T\mathbf{R}^{-1}\mathbf{Z} + \mathbf{G}^{-1})\hat{\mathbf{u}} = \mathbf{Z}^T\mathbf{R}^{-1}\mathbf{y} \tag{1}$$

where $\hat{\boldsymbol{\beta}} = \hat{\theta}$ and $\hat{\mathbf{u}}$ is the column vector of length n containing the $\hat{\gamma}_i$. Then, by direct evaluation, we can see that

1) The matrix $\mathbf{Z}^T\mathbf{R}^{-1}\mathbf{X}$ reduces to the $n \times 1$ column vector where the ith entry is τ^2/σ_i^2.

2) The matrix $(\mathbf{Z}^T\mathbf{R}^{-1}\mathbf{Z} + \mathbf{G}^{-1})$ reduces to the $n \times n$ diagonal matrix with the terms $\tau^2/\sigma_i^2 + 1$ on the diagonal. Hence the matrix $(\mathbf{Z}^T\mathbf{R}^{-1}\mathbf{Z} + \mathbf{G}^{-1})\hat{\mathbf{u}}$ reduces to the $n \times 1$ column vector where the ith entry is $(\tau^2/\sigma_i^2 + 1)\hat{\gamma}_i$.

3) The matrix $\mathbf{Z}^T\mathbf{R}^{-1}$ reduces to the $n \times n$ diagonal matrix with the terms τ^2/σ_i^2 on the diagonal. Hence the matrix $\mathbf{Z}^T\mathbf{R}^{-1}\mathbf{y}$ reduces to the $n \times 1$ column vector where the ith entry is $(\tau^2/\sigma_i^2)y_i$.

Substituting these three matrix expressions into (1), and inspecting the ith entry, gives

$$\frac{\tau^2}{\sigma_i^2}\hat{\theta} + \left(\frac{\tau^2}{\sigma_i^2} + 1\right)\hat{\gamma}_i = \frac{\tau^2}{\sigma_i^2}y_i$$

so that

$$\frac{\tau^2}{\sigma_i^2}\hat{\theta} + \frac{\tau^2(\tau^2 + \sigma_i^2)}{\sigma_i^2\tau^2}\hat{\gamma}_i = \frac{\tau^2}{\sigma_i^2}y_i$$

Dividing this equation by τ^2/σ_i^2 and a little rearrangement gives

$$\hat{\gamma}_i = (1 - B_i)(y_i - \hat{\theta}).$$

where $B_i = \sigma_i^2/(\sigma_i^2 + \tau^2)$, as given in the paper, with the cosmetic difference that we use Y_i instead of y_i.

DERIVATION OF V_i

We write the random-effects model using the variance components representation (see section 2.3 of the paper), so that

$$Y_i = \theta + \gamma_i + \epsilon_i \tag{2}$$

where $\gamma_i \sim N(0, \tau^2)$ and $\epsilon_i \sim N(0, \sigma_i^2)$ and all γ_i and ϵ_i are independent. The true effect of the ith study is

$$\theta_i = \theta + \gamma_i \tag{3}$$

We ignore the uncertainty in τ^2, and all σ_i^2, throughout the derivation that follows. From equation (4) in the paper, and the definition of $\boldsymbol{B}_i$, we have

$$\hat{\theta}_i = \frac{\sigma_i^2}{\sigma_i^2 + \tau^2}\hat{\theta} + \frac{\tau^2}{\sigma_i^2 + \tau^2}Y_i \tag{4}$$

where

$$\hat{\theta} = \sum w_j Y_j / w, \tag{5}$$

$w_j = 1/(\sigma_j^2 + \tau^2)$ and $w = \sum w_j$. We also have the standard result $\text{Var}(\hat{\theta}) = 1/w$.

We calculate

$$V_i = \text{Var}(\hat{\theta}_i - \theta_i) = \text{Var}(\hat{\theta}_i) + \text{Var}(\theta_i) - 2\text{Cov}(\hat{\theta}_i, \theta_i) \tag{6}$$

using the random variables defined in equations (3), (4) and (5). We derive each of the three moments on the right hand side of equation (6) in turn. The first of these moments is

$$\text{Var}(\hat{\theta}_i) = \text{Var}\left(\frac{\sigma_i^2}{\sigma_i^2 + \tau^2}\hat{\theta} + \frac{\tau^2}{\sigma_i^2 + \tau^2}Y_i\right)$$

so that

$$\text{Var}(\hat{\theta}_i) = \left(\frac{\sigma_i^2}{\sigma_i^2 + \tau^2}\right)^2 \text{Var}(\hat{\theta}) + \left(\frac{\tau^2}{\sigma_i^2 + \tau^2}\right)^2 \text{Var}(Y_i) + 2\frac{\sigma_i^2\tau^2}{(\sigma_i^2 + \tau^2)^2}\text{Cov}(\hat{\theta}, Y_i). \tag{7}$$

We have $\text{Var}(Y_i) = \sigma_i^2 + \tau^2 = 1/w_i$, so that

$$\text{Cov}(\hat{\theta}, Y_i) = \text{Cov}(\sum w_j Y_j / w, Y_i) = \frac{1}{w}\text{Cov}(w_i Y_i, Y_i) = 1/w$$

where the second equality in the above equation follows because Y_i is independent of Y_j, so that $\text{Cov}(Y_i, Y_j) = 0$, for $i \neq j$. Hence equation (7) is equal to

$$\text{Var}(\hat{\theta}_i) = \frac{1}{w}\frac{\sigma_i^4}{(\sigma_i^2 + \tau^2)^2} + \frac{\tau^4}{\sigma_i^2 + \tau^2} + \frac{2}{w}\frac{\sigma_i^2\tau^2}{(\sigma_i^2 + \tau^2)^2}$$

which can be simplified so that the first term in the right hand side of (6) is equal to

$$\text{Var}(\hat{\theta}_i) = \frac{\tau^4}{\sigma_i^2 + \tau^2} + \frac{1}{w}\frac{\sigma_i^2(\sigma_i^2 + 2\tau^2)}{(\sigma_i^2 + \tau^2)^2} \tag{8}$$

Equation (8) is equation (13) of the paper.

Trivially the second term in the right hand side of (6) is

$$\text{Var}(\theta_i) = \text{Var}(\theta + \gamma_i) = \text{Var}(\gamma_i) = \tau^2 \tag{9}$$

Equation (9) is equation (14) of the paper.

Finally we derive the third term in the right hand side of (6). From the definition of $\hat{\theta}_i$ in (4) this is

$$-2\text{Cov}(\hat{\theta}_i, \theta_i) = -2\text{Cov}\left(\frac{\sigma_i^2}{\sigma_i^2 + \tau^2}\hat{\theta} + \frac{\tau^2}{\sigma_i^2 + \tau^2}Y_i, \theta_i\right)$$

Upon expanding the right hand side, and substituting equation (5), this is equal to

$$-2\text{Cov}(\hat{\theta}_i, \theta_i) = -2\text{Cov}\left(\frac{\sigma_i^2}{\sigma_i^2 + \tau^2}\sum w_j Y_j / w, \theta_i\right) - 2\text{Cov}\left(\frac{\tau^2}{\sigma_i^2 + \tau^2}Y_i, \theta_i\right)$$

so that

$$-2\mathrm{Cov}(\hat{\theta}_i, \theta_i) = -\frac{2}{w}\frac{\sigma_i^2}{\sigma_i^2 + \tau^2}\mathrm{Cov}\left(\sum w_j Y_j, \theta_i\right) - 2\frac{\tau^2}{\sigma_i^2 + \tau^2}\mathrm{Cov}\left(Y_i, \theta_i\right). \tag{10}$$

We use the variance components definitions of Y_i and θ_i in equations (2) and (3), and the assumption that all γ_i and ϵ_i are independent, to derive

$$\mathrm{Cov}\left(\sum w_j Y_j, \theta_i\right) = \mathrm{Cov}\left(\sum w_j(\theta + \gamma_j + \epsilon_j), \theta + \gamma_i\right) = \mathrm{Cov}\left(w_i\gamma_i, \gamma_i\right) = \frac{\tau^2}{\sigma_i^2 + \tau^2} \tag{11}$$

and

$$\mathrm{Cov}\left(Y_i, \theta_i\right) = \mathrm{Cov}\left(\theta + \gamma_i + \epsilon_i, \theta + \gamma_i\right) = \mathrm{Cov}\left(\gamma_i, \gamma_i\right) = \tau^2. \tag{12}$$

Substituting equations (11) and (12) into (10) gives

$$-2\mathrm{Cov}(\hat{\theta}_i, \theta_i) = -\frac{2}{w}\frac{\sigma_i^2\tau^2}{(\sigma_i^2 + \tau^2)^2} - \frac{2\tau^4}{\sigma_i^2 + \tau^2}. \tag{13}$$

Equation (13) is equation (15) of the paper.

How to cite this article: , , , , and (), , , .
