# Supplementary figures and images for: Study specific prediction intervals for random‐effects meta‐analysis: A tutorial: Prediction intervals in meta‐analysis
Source: Res Synth Methods. 2021 Jun 3;12(4):429–47. doi: 10.1002/jrsm.1490 (PMC8361666; doi:10.1002/jrsm.1490)

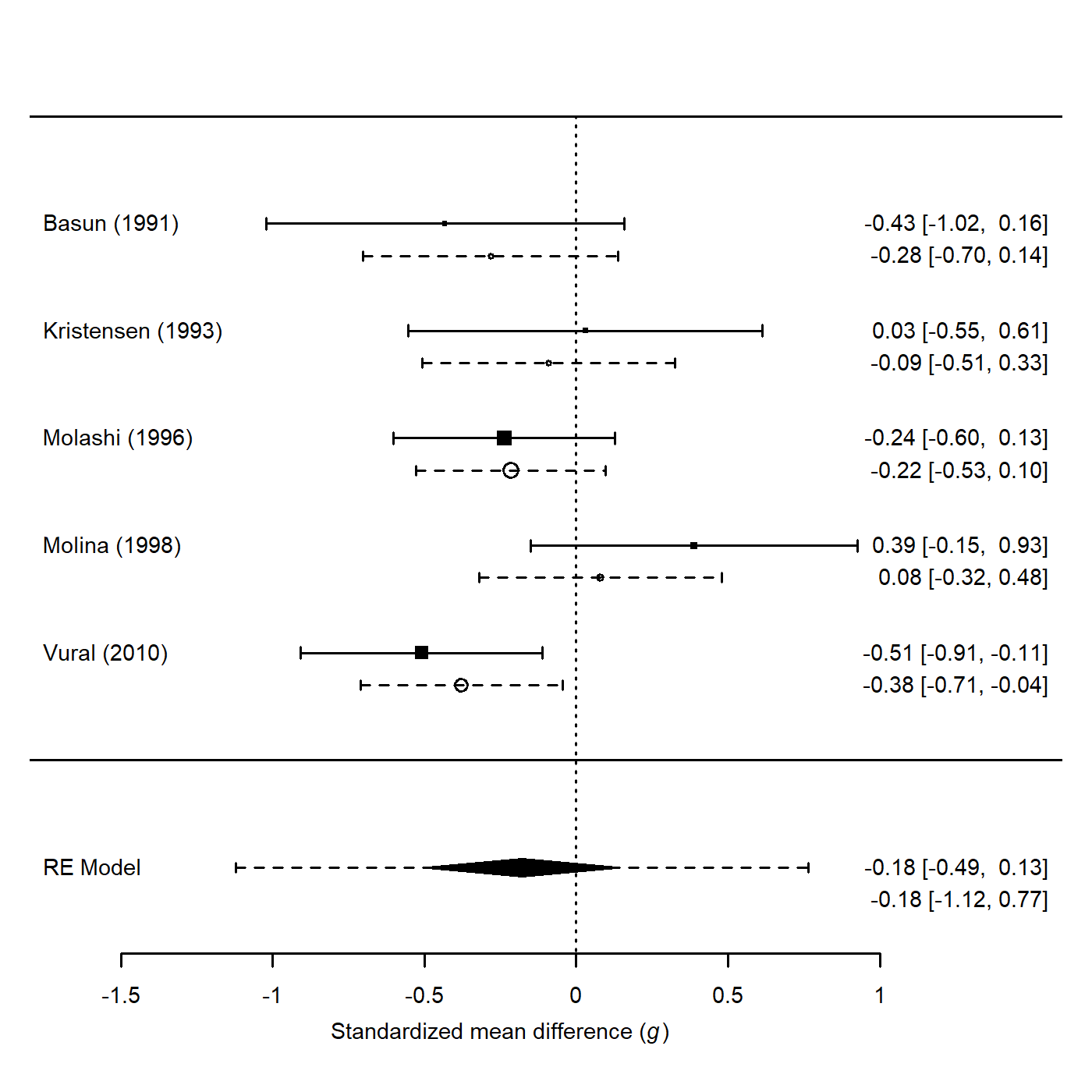

Supplement: Supplementary file 1 — AppendixS1. Supplementary Information [file JRSM-12-429-s001.zip › JRSM_1490_Blups_alz.png]

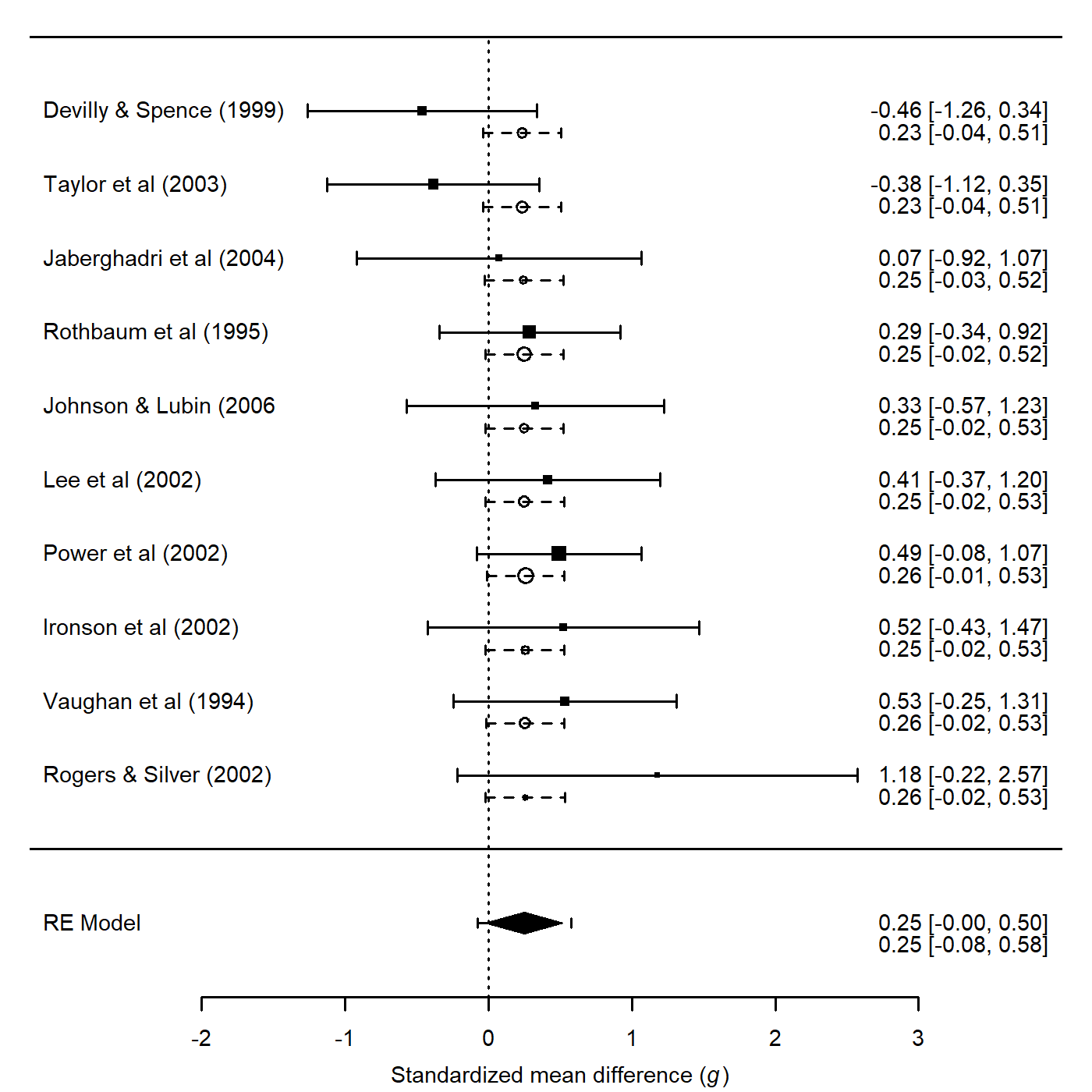

Supplement: Supplementary file 1 — AppendixS1. Supplementary Information [file JRSM-12-429-s001.zip › JRSM_1490_Blups_PTSD.png]

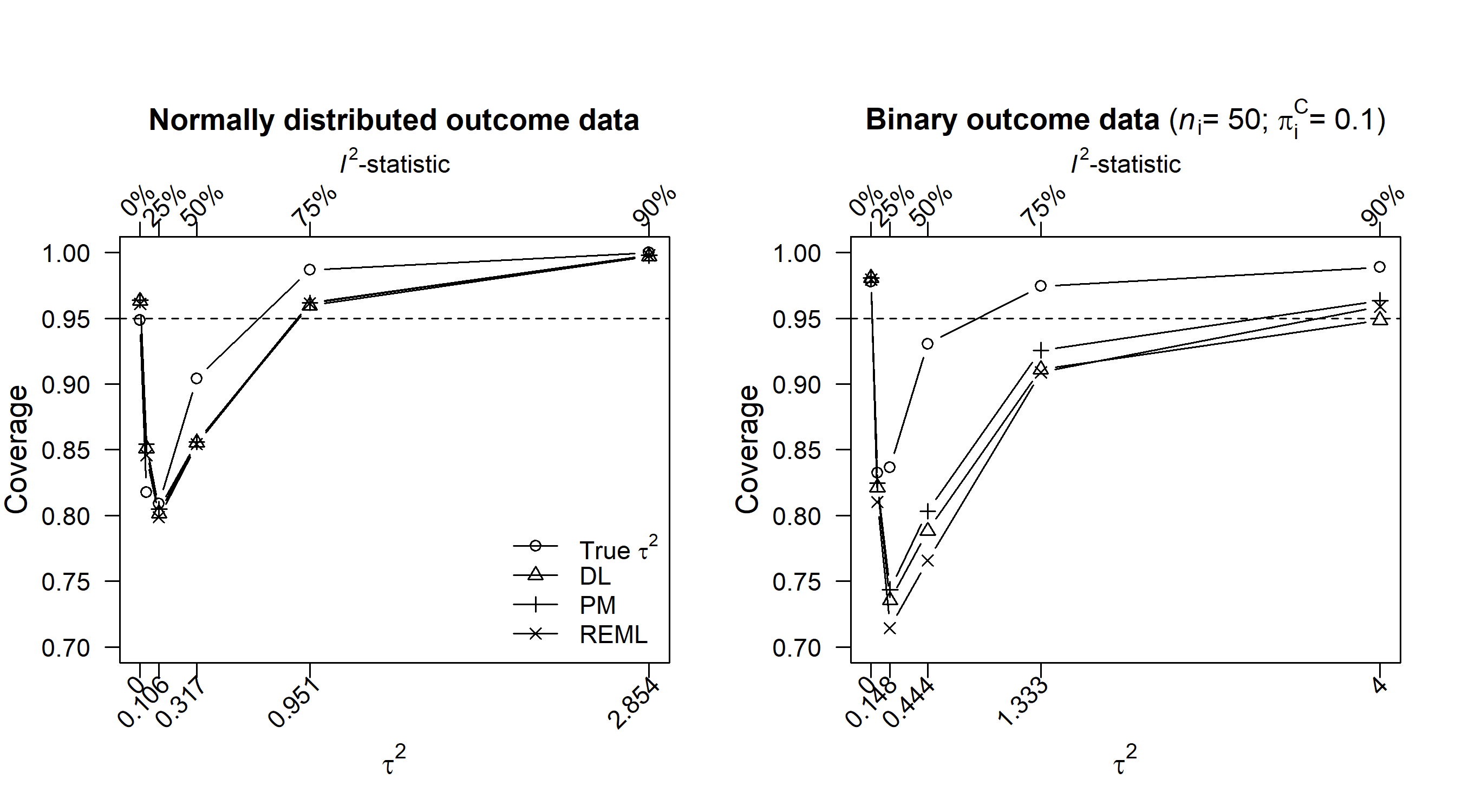

Supplement: Supplementary file 1 — AppendixS1. Supplementary Information [file JRSM-12-429-s001.zip › JRSM_1490_figs_quan_n50.png]

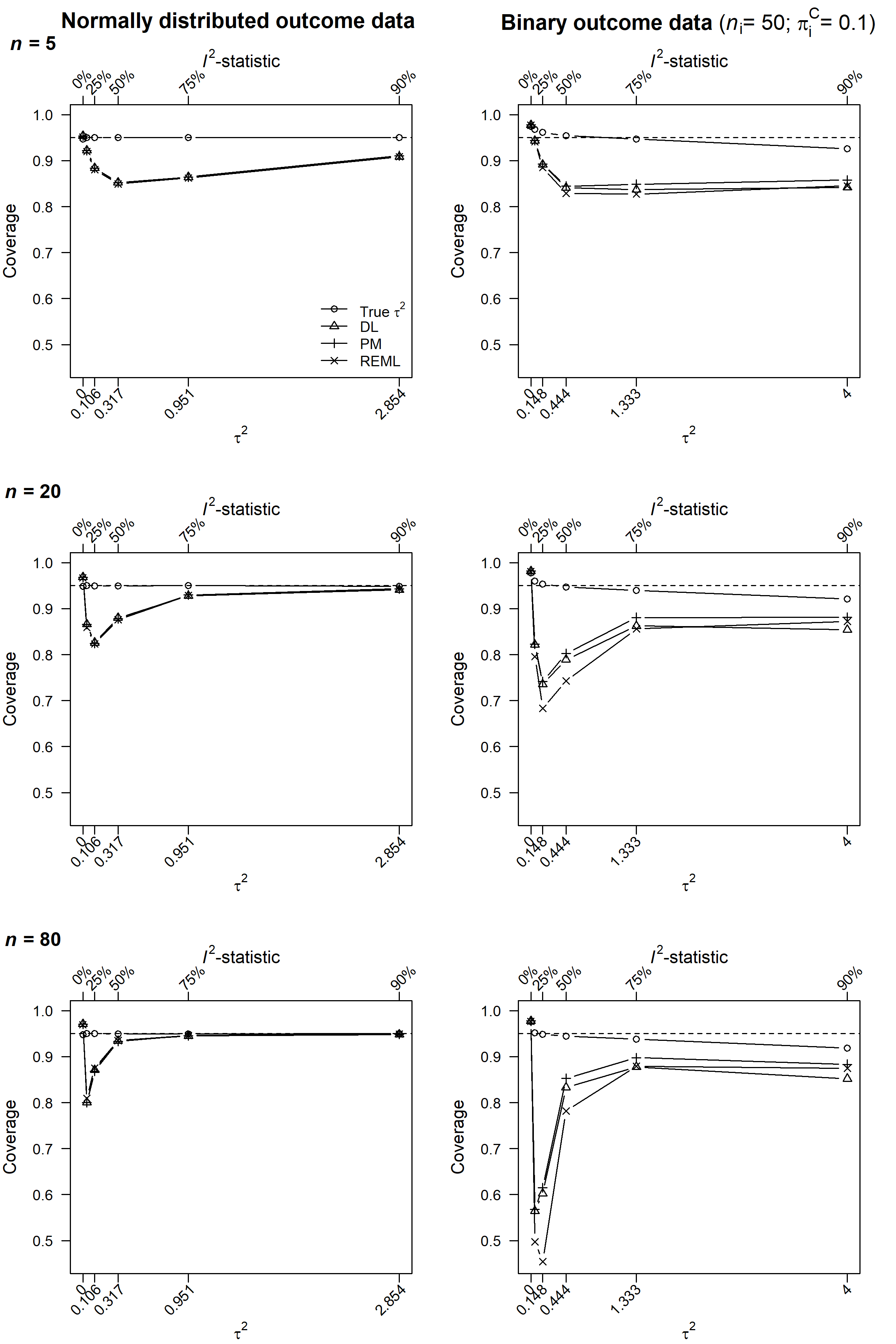

Supplement: Supplementary file 1 — AppendixS1. Supplementary Information [file JRSM-12-429-s001.zip › JRSM_1490_figs_sim_n50.png]
